# Conservation and Divergence of *SQUAMOSA-PROMOTER BINDING PROTEIN-LIKE* (*SPL*) Gene Family between Wheat and Rice

**DOI:** 10.3390/ijms23042099

**Published:** 2022-02-14

**Authors:** Li Li, Fu Shi, Guoli Wang, Yanbin Guan, Yufan Zhang, Mingjie Chen, Junli Chang, Guangxiao Yang, Guangyuan He, Yuesheng Wang, Yin Li

**Affiliations:** The Genetic Engineering International Cooperation Base of Chinese Ministry of Science and Technology, Key Laboratory of Molecular Biophysics of Chinese Ministry of Education, College of Life Science and Technology, Huazhong University of Science and Technology, Wuhan 430074, China; hustll2017@hust.edu.cn (L.L.); hustshifu@hust.edu.cn (F.S.); m202071947@hust.edu.cn (G.W.); gyb@hust.edu.cn (Y.G.); d201980545@hust.edu.cn (Y.Z.); cmj@hust.edu.cn (M.C.); cjl@hust.edu.cn (J.C.); ygx@hust.edu.cn (G.Y.) ; hegy@hust.edu.cn (G.H.)

**Keywords:** conservation and divergence, expression patterns, genome-wide analysis, *SQUAMOSA-PROMOTER BINDING PROTEIN-LIKE* (*SPL*) gene, *Triticum aestivum*, *Oryza sativa*

## Abstract

The *SQUAMOSA-PROMOTER BINDING PROTEIN-LIKE* (*SPL*) gene family affects plant architecture, panicle structure, and grain development, representing key genes for crop improvements. The objective of the present study is to utilize the well characterized *SPL*s’ functions in rice to facilitate the functional genomics of *TaSPL* genes. To achieve these goals, we combined several approaches, including genome-wide analysis of *TaSPL*s, comparative genomic analysis, expression profiling, and functional study of *TaSPL3* in rice. We established the orthologous relationships of 56 *TaSPL* genes with the corresponding *OsSPL*s, laying a foundation for the comparison of known *SPL* functions between wheat and rice. Some *TaSPL*s exhibited different spatial–temporal expression patterns when compared to their rice orthologs, thus implicating functional divergence. *TaSPL2*/*6*/*8*/*10* were identified to respond to different abiotic stresses through the combination of RNA-seq and qPCR expression analysis. Additionally, ectopic expression of *TaSPL3* in rice promotes heading dates, affects leaf and stem development, and leads to smaller panicles and decreased yields per panicle. In conclusion, our work provides useful information toward cataloging of the functions of *TaSPL*s, emphasized the conservation and divergence between *TaSPL*s and *OsSPL*s, and identified the important *SPL* genes for wheat improvement.

## 1. Introduction

Wheat is one of the most important crops worldwide, providing a food supply for about 28% of the global population [1]. However, sustaining wheat yield and quality has become unprecedentedly challenging for several reasons, including the reduction of arable land area, water resource shortages, and the emergence of new pathogens and pests. Our fundamental understanding of the genes involved in wheat functional traits represents one of the key aspects for wheat molecular breeding and, hence, is of great significance for the improvement of wheat yield and quality.

Transcription factors (TFs) represent a particular type of DNA-binding proteins encoded by certain gene families, which bind to their target genes in a sequence-specific manner and activate or inhibit the transcription of target genes. Thus, TF families are involved in various aspects of plant growth and development, and often contain master regulators and key genes for crop improvement.

Among the many important TF families, the SQUAMOSA-PROMOTER BINDING PROTEIN-LIKE (SPL) family is a plant-specific family that has expanded to play a diverse variety of functions during plant evolution, involving shoot architecture [2], axillary bud formation [3], plant architecture [4,5,6], male sterility [7], flowering regulation [8], inflorescence branching [9,10], organ size [11,12,13,14], and grain development [8,11]. A typical SPL protein has a 78 amino-acid SQUAMOSA-PROMOTER BINDING PROTEIN (SBP) domain, which is highly conserved across SPL family members [15,16]. This domain contains a novel zinc finger motif with two Zn^2+^ binding sites—Cys-Cys-His-Cys (CCHC) and Cys-Cys-Cys-His (CCCH)—and a nuclear localization signal (NLS) [15]. This domain can specifically bind to the cis-element TNCGTACAA at a gene promoter region for regulation of SPL target genes [17,18].

*SPL* genes (*AmSBP1* and *AmSBP2*) were first found to regulate early flowering in *Antirrhinum majus* L. [19]. Since then, the SPL family has been characterized in many important species, including the identification of 17 *AtSPL* genes in Arabidopsis, 19 *OsSPL* genes in rice, 31 *ZmSPL* genes in maize, 15 *SlSPL* genes in tomato, 27 *MdSPL* genes in apple, and 18 *VvSPL* genes in grape [15,20,21,22,23,24].

In the model plant Arabidopsis, the functions of some At*SPL*s have been well-studied. Overexpression of *AtSPL3* in transgenic *Arabidopsis thaliana* (L.) resulted in a shorter vegetative growth period and earlier flowering [18]. *AtSPL4* and *AtSPL5* play a similar function as *AtSPL3* [18,25,26]. Deficient *AtSPL8* function in an Arabidopsis mutant led to floral infertility due to the abnormal development of microsporangiums and macrospores [27]. A functional deficiency of *AtSPL10* brought about an increase of lateral branch number; *AtSPL2* and *AtSPL11* had similar phenotypic effects [28]. A loss of *AtSPL14* function led to the elongation of leaf petioles, enhancement of leaf margins, and better plant resistance to the fungal toxin FB1 [29].

In rice, almost every member of *OsSPL* has been functionally characterized by transgenic, mutant, or genome-editing approaches [5,6,11,13,14,30,31,32,33,34,35,36,37]. A knockout of *OsSPL3* gene in rice has been observed to delay head date, as well as changing the panicle structure [31]. After *OsSPL6* gene knockout in rice, the length of plant inflorescences was shortened, and the number of inflorescence branches was decreased [36]. Overexpression of *OsSPL7* in rice decreased tiller numbers, possibly through the miR156F-OsSPL7-OSGH3.8 pathway [30]. A loss of the *OsSPL8* gene in a rice mutant has been characterized by a complete loss of auricles, ligules, and laminar joints and, hence, erect leaves [32]. In overexpressed *OsSPL9* transgenic rice, increased Cu content in mature grains has been observed, with the expression of Cu transporter genes COPT1, COPT5, and COPT6 being upregulated in these rice seedlings [34]. *OsSPL12* has been shown to regulate the root development of rice, through the OsmiR156-OsSPL12-OsMADS50 pathway [33]. *OsSPL13* regulates the cell size of spikelet hulls in rice, thus altering grain length and weight [11]. *OsSPL14* overexpression in transgenic rice has been confirmed to decrease ineffective tillers, enhance stem strength, increase inflorescence branch numbers, and induce immunity to fungal infection, hence promoting grain yields and conferring the ideal plant architecture (IPA) of rice [5,6,35]. Additionally, the loss of *OsSPL16* function in rice resulted in slender grains and reduced the weight per thousand grains [13,14]. *OsSPL18* showed a similar function [37]. More importantly, almost every member of *OsSPL* has been functionally characterized in rice by a transgenic, mutant, or genome-editing approach, demonstrating not only their functional redundancy but also sub-functionalization in paleoduplicated *OsSPL* pairs [31]. These results emphasize the strong impact of the most recent whole-genome duplication (WGD), which occurred approximately 70–90 million years ago (MYA), on the expansion of the *SPL* family and its evolutionary innovation.

Different from the diploid genome of rice, common wheat (*Triticum aestivum*) has an allopolyploid genome (AABBDD, 2*n* = 6*x* = 42), with its A, B, and D sub-genomes originating from the wild diploid species *Triticum urartu* (AA, 2*n* = 2*x* = 14), *Aegilops speltoides* (BB, 2*n* = 2*x* = 14), and *Aegilops tauschii* (DD, 2*n* = 2*x* = 14), respectively [38,39,40]. Therefore, the *TaSPL* functions may have been impacted by several evolutionary aspects, including paleoduplication, allopolyploidization, and tandem or segmental duplication of genes. On one hand, several gene family studies have consistently identified 56 *TaSPL* genes in wheat [41,42,43,44]. On the other hand, functional characterization of *TaSPLs* in transgenic monocot plants remains very limited [45,46,47]. For example, transgenic wheat lines with *TaSPL8* gene edited by the CRISPR (clustered regularly interspaced short palindromic repeats)/Cas9 method were characterized by deficient leaf stalk bases (auricles, ligules, and laminar joints), erect leaves, and compact plant architectures [46]. Overexpression of *TaSPL13* affects inflorescence architectures in wheat, with an increased number of florets and grains [45]. Other *TaSPL*s have been functionally studied in ectopic expression systems, including rice and Arabidopsis. In transgenic rice, panicle length, primary, and secondary branches of the panicle, and grain numbers were increased significantly after overexpression of *TaSPL20* and *TaSPL21* (renamed to *TaSPL10* and *TaSPL5*, which are actually the wheat orthologs of *OsSPL10* and *5*, respectively) [47]. Ectopic overexpression of *TaSPL16* in *A. thaliana* promoted flowering [48], while *TaSPL3* or *TaSPL6* overexpression in *A. thaliana* affected flowering time and organ size [49].

As techniques for the generation of transgenic or genome-edited plants are still not routine in wheat, and are challenging and time-consuming, utilizing the existed knowledge of *OsSPL* functions is expected to facilitate the functional study of *TaSPL*s. We hypothesize that a combination of the spatial–temporal expression patterns of *TaSPL*s, the orthologous relationships between *TaSPL*s and *OsSPL*s will greatly help to understand and to predict the functions of *TaSPL*s. We also hypothesize that a *TaSPL* gene may have largely conserved or overlapping functions with the *OsSPL* ortholog if both the *OsSPL* and *TaSPL* exhibit a similar expression pattern. For the present study, we aimed to: (1) establish orthologous relationships between *TaSPL*s and *OsSPL*s; (2) collect expression evidence for the functional conservation and divergence of *TaSPL* members; and (3) summarize the current state-of-the-art of *OsSPL* functions and to identify and validate examples of *TaSPL* genes with conserved functions between wheat and rice.

## 2. Results

During the evolution of *Brassicaceae* and *Pooideae*, Arabidopsis and rice have experienced distinct sets of paleopolyploidization events, which have had major impacts in terms of gene expansion and functional divergence. In light of this, *SPL*s have expanded in a lineage-specific manner in Arabidopsis and rice, even though Arabidopsis and rice have similar numbers of *SPL* genes (17 in Arabidopsis vs. 19 in rice) [15,23]. Differing from *AtSPL*s, lineage-specific gene expansion has involved *OsSPL*s in a diverse set of biological processes related to plant growth and development, which have been functionally characterized using either mutants, transgenic lines, or CRISPR/Cas9-based knockout lines [5,10,11,13,14,31,37,50,51]. Among the agronomically important monocot cereal crops, rice and wheat are very similar in plant development and architecture, except for their inflorescence structures, making it possible to transfer the current knowledge of gene functions from rice to wheat.

To utilize the large volume of knowledge related to the functions of *OsSPLs*, we combined BLAST- and protein domain-based methods for gene identification and found 56 genes encoding TaSPLs. The *TaSPL*s reported in our work are consistent with those identified in several previous studies [41,44,45]. All of the TaSPL proteins have one SBP domain with a length of approximately 78 amino acids, and which is conserved between OsSPLs and TaSPLs (Figure S1). This SBP domain contains two zinc-binding sites—the zinc finger 1 motif and zinc finger 2 motif—together with a conserved nuclear localization signal (NLS) located in the C-terminal of the SBP domain. TaSPLs have a Cys-Cys-Cys-His (CCCH)-type zinc finger 1 motif, except for TaSPL9-A/B/D, which contain the Cys-Cys-Cys-Cys (CCCC) type, while all TaSPLs have the Cys-Cys-His-Cys (CCHC)-type zinc finger 2 motif (Figure S1).

### 2.1. Polyploidization and Gene Tandem Duplication Shapes the Expansion of TaSPLs

**Figure 1:**
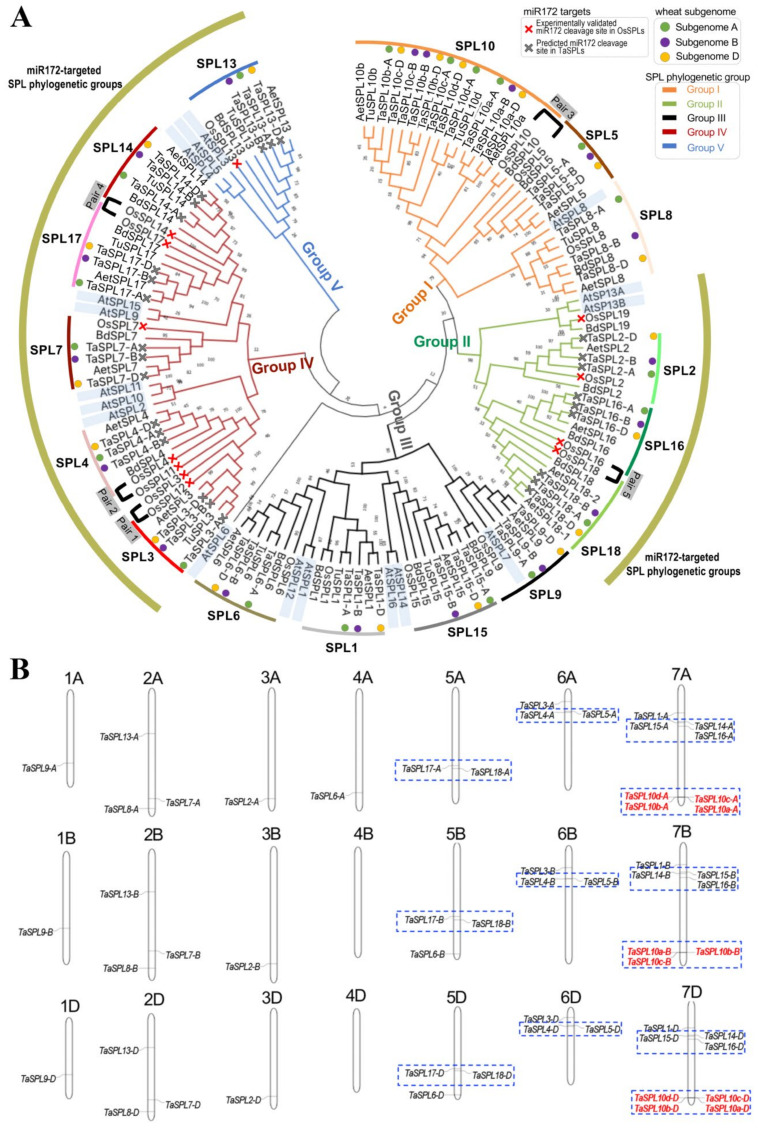
Phylogenetic analysis of *TaSPL*s highlighting the orthologous relationship between *OsSPL*s and *TaSPL*s: (**A**) Phylogenetic tree constructed by maximum-likelihood method grouped *TaSPL*s together with their orthologs in rice, providing results consistent with the syntenic analysis of *SPL* genes between rice and wheat (Figure S2, Table S1); (**B**) diagram of the chromosomal locations of *TaSPL* genes, identifying several *TaSPL* clusters and gene expansion at the *TaSPL10* locus driven by tandem duplications. *TaSPL* gene clusters are shown by blue boxes and the tandemly duplicated genes of *TaSPL10* are highlighted in red.

To focus on the phylogeny of SPLs in Pooideae, we combined the TaSPLs with 18 SPLs from *Aegilops tauschii* (*Ae. tauschii*), 10 SPLs from *Tritucum urartu* (*T. urartu*), 16 SPLs from *Brachypodium distachyon* (*B. distachyon*), and the 19 SPLs from *Oryza sativa* L. (*O. sativa*) to construct a maximum-likelihood tree (Figure 1) [41,44]. Phylogenetic analysis clustered the SPLs into five groups (groups I–V). Further, we compared the micro-synteny between the genomic segments harboring OsSPL genes and TaSPL genes in order to determine the wheat syntelogs of each OsSPL gene (Figure S2, Table S1). Consistent with the syntelogous relationship between OsSPLs and TaSPLs, our phylogenetic tree clustered each set of TaSPL homeologs well (labeled using the green, purple, and yellow circles for the homeologous copies from the wheat A, B, and D sub-genomes, respectively), together with the corresponding rice ortholog (Figure 1A). In rice, among the 19 OsSPLs, ten genes (accounting 52% of the OsSPL family) formed five sister gene pairs, which are hereafter named SPL pairs 1 to 5 for OsSPL3/12, OsSPL4/11, OsSPL5/10, OsSPL14/17, and OsSPL16/18, respectively, in order to describe the SPL genes in the context of evolution. These five paleoduplicated gene pairs were retained after ancient whole-genome duplication (WGD) events, with evidence of sub-functionalization [31]. In contrast to OsSPLs, deletions of pair-1 and pair-2 SPLs (SPL3/12 and SPL4/11) occurred in wheat and its diploid relative species, retaining only SPL3 and SPL4, respectively (Figure 1). More interestingly, the tandem duplications have driven TaSPL10 expansion (belonging to the pair-3 SPL), resulting in 11 copies, designated as TaSPL10a-A/B/D, TaSPL10b-A/B/D, TaSPL10c-A/B/D, and TaSPL10d-A/D (Figure 1). For singleton SPLs, syntelogous relationships have been well-kept between OsSPLs and TaSPLs, indicative of the possibly different evolutionary fates of singleton SPLs and paleoduplicated SPL pairs in wheat. In addition, SPL19 genes were not identified in the analyzed Triticeae species. Taken together, our genome-wide analysis revealed that the expansion of TaSPL genes in wheat are a consequence of the allopolyploidization of A/B/D sub-genomes and the tandem duplication of TaSPL10.

A gene cluster is a group of specific genes with close spatial proximity (up to 10 Mb, depending on the species) on a chromosomal segment [52]. The ten paired *OsSPL*s belonged to four *SPL* gene clusters: *OsSPL3/4/5*, *OsSPL12/11/10*, *OsSPL14/15/16*, and *OsSPL17/18* on rice chromosomes 2, 6, 8, and 9, respectively [31]. Due to the deletion of *TaSPL11/12*, only tandemly duplicated copies of *TaSPL10* exist on the corresponding segments in wheat chromosome 7 (Figure 1B), while the other clusters of *TaSPL* genes (cluster 1 *TaSPL3/4/5*, cluster 3 *TaSPL14/15/16*, and cluster 4 *TaSPL17/18*) have larger distances between genes from the same cluster (Figure 1B), likely due to chromosomal rearrangements during the evolution and expansion of the wheat genome [53,54].

Analyses of gene structures and protein motifs found, on one hand, that *TaSPLs* from the same phylogenetic groups tend to have similar exon–intron structures and similar combinations of MEME-predicted protein motifs (Figure S3). On the other hand, differences in the predicted protein motifs between *SPL* triads were sometimes observed, suggesting divergence between *SPL* triads at the protein sequence or protein–protein interaction levels (shown in red boxes in Figure S3B).

MicroRNA156 (miR156) is the major microRNA that regulates several *SPL* genes at the post-transcriptional level in many plant species [55]. The miR156-*SPL* is an important regulatory module for plant growth and development [3,5,26,56,57,58]. In rice, 12 of the 19 *OsSPL*s are targeted by miR156, covering phylogenetic groups II, IV, and V of *OsSPL*s (labeled by red crosses in Figure 1A). As the miRNA–target complementary rules in plants have been well studied [59], we utilized psRNATarget prediction to identify the *TaSPL*s that are likely targeted by miR156 (Figure S4) [60]. A total of 27 *TaSPL*s (i.e., the TaSPL2, 3, 4, 7, 13, 14, 16, 17, and 18 triads) were predicted to be targeted by tae-miR156 (Figure S4), all of which are evolutionarily conserved wheat synteologs of the miR156-regulated *OsSPL*s (Figure 1A). Sequence alignment results demonstrated that the mature tae-miR156 sequence is conserved between dicot and monocot species, while the miR156 recognition sites in *TaSPL*s have a few sequence variations between *TaSPL* genes (Figure S5), suggesting that miR156-*SPL* regulation is likely conserved between rice and wheat.

### 2.2. Expression Profiling Indicates the Conservation and Divergence of TaSPL Genes

**Figure 2:**
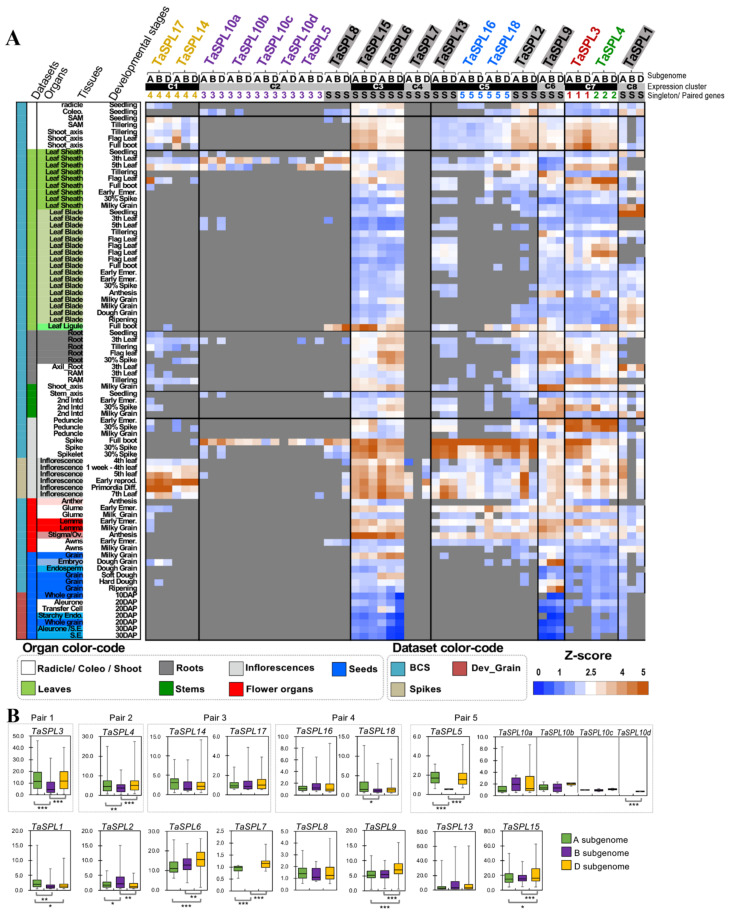
Expression analyses of *TaSPL*s emphasizing the divergence of some *TaSPL*s, in terms of spatial–temporal expression patterns: (**A**) RNA-seq based expression of *TaSPL*s. Each column represents a *TaSPL* gene, and each row represents an RNA-seq sample, with the RNA-seq data sets, tissues, and stages labeled on the left. A, B, D indicate the sub-genome that each *TaSPL* gene is located on. In Figure 1A, singleton *TaSPL*s are shaded in gray, while evolutionarily paired *TaSPL*s are highlighted using colors, with red, green, gold, blue, and purple indicating Pair 1, Pair 2, Pair 3, Pair 4, and Pair 5, respectively. The *TaSPL* genes are row-clustered into eight clusters (namely, C1–C8) based on their expression similarity, as determined by the k-mean clustering method; (**B**) comparison of expression between each set of *TaSPL* homeologous copies, identifying multiple *TaSPL* genes with sub-genome expression biases. The Y-axis indicates the gene expression levels (as TPM). Statistical significance of sub-genome expression biases (SEB) for each *TaSPL* gene was determined by two-tailed Student’s *t*-test, with *, **, and *** representing *p* < 0.05, *p* < 0.01, and *p* < 0.005, respectively (details in Method Section 4.5).

Determining when and where a gene is expressed is a necessary step to perform reverse genetics for the study of gene functions. Several RNA-seq studies have recently been reported based on the high-quality wheat reference genome, documenting gene expression profiles in various tissues and organs across wheat developmental stages [61]. We utilized RNA-seq data and compiled expression profiles of *TaSPL*s in leaves, roots, stems, inflorescences, flowers, and seeds (Figure 2A), likely representing one of the most comprehensive expression analyses of *TaSPL*s, to the best of our knowledge.

#### 2.2.1. RNA-Seq Analysis Highlights Sub-Genome Expression Biases of TaSPL Genes

*TaSPL* genes were hierarchically clustered into seven clusters (namely, Clusters 1–7), based on their RNA-seq expression patterns (Figure 2A). Clusters 1, 2, and 4 (containing *TaSPL14/17*, *TaSPL5/10,* and *TaSPL7*, respectively) are those with tissue-specific expression patterns, while Cluster 5 (*TaSPL2/13/16/18*) contains *TaSPL*s preferentially expressed in some tissues or developmental stages. The remaining *TaSPL*s (Clusters 3, 6, 7, and 8; see Figure 2A) were widely expressed in multiple tissues. For instance, *TaSPL14/17* were strongly expressed in shoots and inflorescences. All of the *TaSPL10* copies were specifically expressed in young leaves and spikes. Moreover, we found that *TaSPL8* exhibited strong and specific expression in the leaf ligule, matching its validated function in leaf ligule development [46]. In addition, *TaSPL1/3/4/6/9/15* were widely expressed in multiple tissues, with strong expression observed in developing tissues and organs, including shoot apical meristem (SAM), shoots and developing roots, stems, inflorescences, and seeds, indicating that these *TaSPL*s might play pleiotropic roles in the regulation of plant development. Meanwhile, we validated the expression profiles of several *TaSPL*s, including *TaSPL2/3/4/6/8/10/17/18*, in wheat *cv*. Chinese Spring at multiple development stages and in various tissues (Figure S6).

Our RNA-seq analysis demonstrated a good agreement between the expression patterns and biological functions of TaSPLs. For example, *TaSPL13* was predominantly expressed in inflorescences (Figure 2A), consistent with the qPCR-based spatial–temporal expression results, matching its confirmed functions in spike and floret development in wheat [45]. *TaSPL3* and *TaSPL6* had similar expression patterns, with highest expression observed in the shoot axis and spikes at boot stage (Figure 2A), in agreement with the functions in regulating heading date and flowering time in *A. thaliana* [49].

These expression results also indicated potential functional divergence between the paleoduplicated *TaSPL* gene pairs (i.e., *TaSPL16* and *TaSPL18*). For example, *TaSPL18* was expressed in the leaf sheath across multiple stages, while *TaSPL16* was not; furthermore, *TaSPL18* showed stronger expression levels in flower organs (glume, lemma, and stigma) than *TaSPL16*. In contrast, the other two duplicated pairs of *TaSPL*s (*TaSPL5/10* and *TaSPL14/17*) showed relatively similar expression profiles.

As a polyploid species, the homeologous copies from the three sub-genomes of common wheat adopted different spatial–temporal expression patterns or were expressed at distinct levels, thus differing in their contributions to a particular phenotype. This phenomenon is known as sub-genome expression bias (SEB). We analyzed the SEBs of *TaSPL*s using RNA-seq data. Statistical analysis of the expression ranges between the A, B, and D sub-genome *TaSPL* homeologous copies revealed 11 *TaSPL* genes with SEBs. Particularly, many *TaSPL*s (*TaSPL1/3/4/5/9/15/18*) from the B sub-genome had relatively lower expression levels, when compared with the corresponding copies from A and D sub-genomes (Figure 2B). Typical examples are as follows: *TaSPL7-B* was not expressed in any of the analyzed RNA-seq samples, while *TaSPL7-A* and *-D* were expressed in inflorescences; furthermore, *TaSPL1-B* was not expressed in most shoot, leaf sheath, and inflorescence samples, while *TaSPL1-A* and *-D* were expressed, indicating potential sub-functionalization of *TaSPL1-B*.

#### 2.2.2. RNA-Seq Analysis Shows the Different Spatial–Temporal Expression Preferences between TaSPLs and OsSPLs

**Figure 3:**
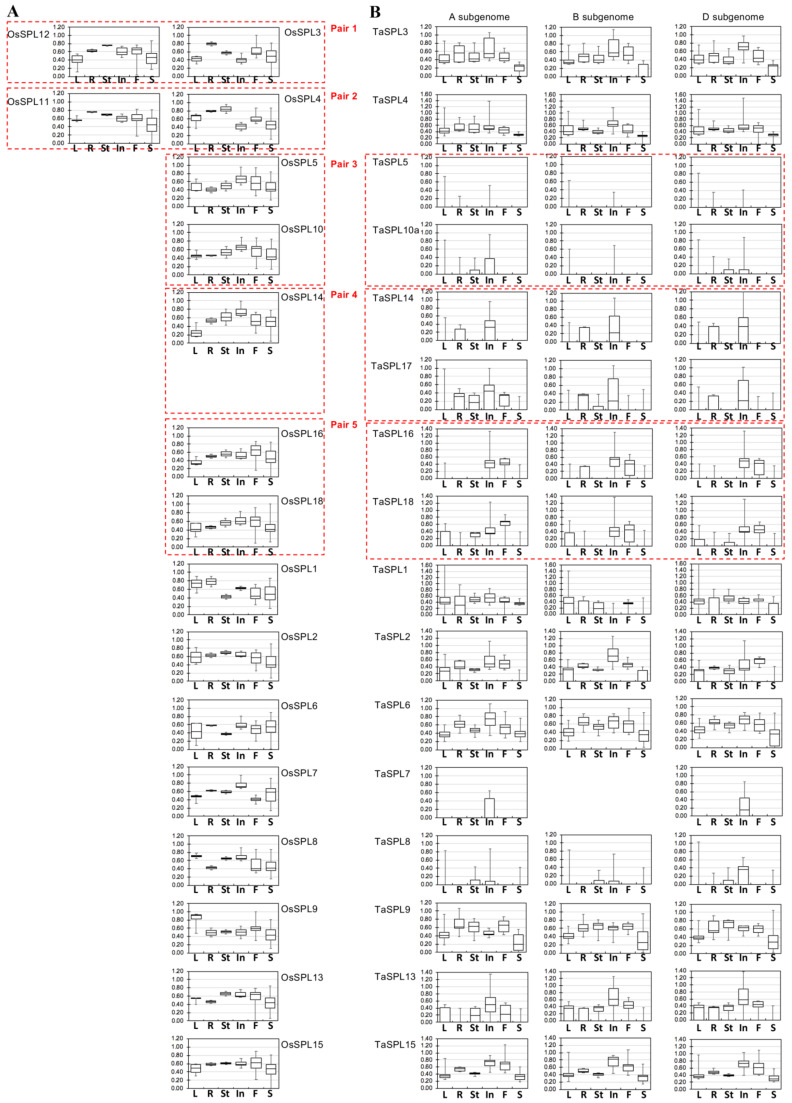
Summarized expression patterns of *OsSPL*s (**A**) and their orthologous *TaSPL*s (**B**). The *SPL* genes are listed first by paleoduplicated pairs and then singletons, with the duplicated pairs of *SPL*s indicated in red boxes. *OsSPL* expression profiles are from microarray data (Figure S7) and summarized in terms of tissues (L: leaves; R: roots; St: stems; In: inflorescence; F: flower organs; S: seed tissues), while the *TaSPL* expression profiles were retrieved from publicly available RNA-seq results (details in Methods section).

With the extensive expression data of *TaSPL*s, we questioned whether the wheat *SPL* orthologs retained similar expression preferences after rice–wheat divergence. To address this, we retrieved both the microarray- and RNA-seq-based expression profiles of *OsSPL*s (Figure S7). The microarray expression data of *OsSPL*s cover a wide range of tissues/organs across different rice developmental stages [23], with comparable tissues and stages to the wheat expression atlas of cv. Azhurnaya [62] (see Method Section 4.5). We summarized the *TaSPL* and *OsSPL* expression profiles, respectively, for each organ (i.e., leaves, roots, stems, inflorescences, flowering organs, and seed tissues), in order to simplify the comparison between rice and wheat (Figure 3). Both *TaSPL*s and *OsSPL*s can be grouped into three classes—namely, ubiquitously expressed, tissue-preferentially expressed, and specifically expressed—based on the number of tissues where each *SPL* gene is expressed (see Methods section). In rice, *OsSPL1/3/4/6/9/11/12/15* are ubiquitously expressed, *OsSPL2/8/14/16/18* are expressed preferentially in some tissues, and *OsSPL5/7/10/13/17* are expressed specifically in particular tissues (Figure S7). In wheat, *TaSPL1/3/4/6/9/15* are ubiquitously expressed, *TaSPL2/13/16/18* have tissue-preferential expression patterns, and *TaSPL5/7/8/10/14/17* are expressed in a tissue-specific manner (Figure 2A). The *SPL* genes with ubiquitous and tissue-preferential expression largely overlap in rice and wheat, whereas the tissue-specifically expressed *SPL* genes mostly differed between rice and wheat.

In rice, the two *SPL* genes within the paleoduplicated pairs 1 or 2 showed distinct expression patterns; for example, *OsSPL3* had highest expression in roots, whereas *OsSPL12* was highly expressed in roots, stems, inflorescences, and flower organs (Figure 3A). In contrast, the two *OsSPL* genes within each duplicated pair (e.g., *OsSPL5/10* and *OsSPL16/18*) exhibited similar expression abundance at the organ level.

In wheat, the majority of *SPL* triads showed similar organ-level expression preferences (Figure 3B); however, *TaSPL1-B* had relatively lower expression in roots, stems, and flower organs, when compared with those of *TaSPL1-A* and *-D*. *TaSPL18-A* and *-D,* but not *TaSPL18-B,* were expressed in stems. It has been well-accepted that where a gene is expressed is correlated to its function in a particular tissue/organ. Therefore, we considered that comparison of the expression patterns at tissue or organ levels between *OsSPL*s and *TaSPL*s provides valuable information regarding the transfer of the known functions of *OsSPL*s to *TaSPL*s, thus facilitating the functional study of *TaSPL*s. For example, *TaSPL8* and *OsSPL8* have high expression levels specifically in the leaf sheath and ligule; indeed, their conserved function in regulating the development of the leaf ligule can be reasoned from such expression analyses, and has been experimentally confirmed [32,46].

When comparing the expression patterns of *TaSPLs* and *OsSPL*s, some *TaSPL*s appeared to exhibit expression patterns differing from their rice *SPL* orthologs, with some *TaSPL*s even being expressed in a manner much more specific to certain tissues and stages. For instance, *OsSPL7* is highly expressed in both inflorescences and seeds, while *TaSPL7* is specifically expressed in inflorescence tissues (Figure 3, Figure S7). Another example is that *OsSPL5* is expressed highly in inflorescences, flower organs, and seeds, whereas *TaSPL5* is particularly expressed in leaf, root, and inflorescence samples. These obvious changes in expression patterns between *OsSPLs* and their orthologs in wheat possibly indicate the *TaSPL*s may possess functions different from those of the corresponding *OsSPL*s.

#### 2.2.3. TaSPLs Respond to Abiotic Stresses and Phytohormone Treatments

While *TaSPLs* exhibited spatial–temporal expression preferences, the potential roles of *TaSPL*s in stress response and regulation have been understudied. This is, at least partly, due to the fact that the high-quality wheat reference genome has only become available recently, and comprehensive transcriptome studies of wheat plants under stress treatments have been limited. Based on the limited RNA-seq data of different wheat varieties treated by abiotic stresses (summarized in Table S3) [63,64], we selected the stress-responsive *TaSPL* genes to perform qPCR expression analysis under abiotic stresses and phytohormone treatments, in order to gain more insights into the roles of *TaSPL*s in stress responses and regulation, as many stress-tolerance mechanisms are mediated by phytohormone signaling pathways [65,66].

**Figure 4:**
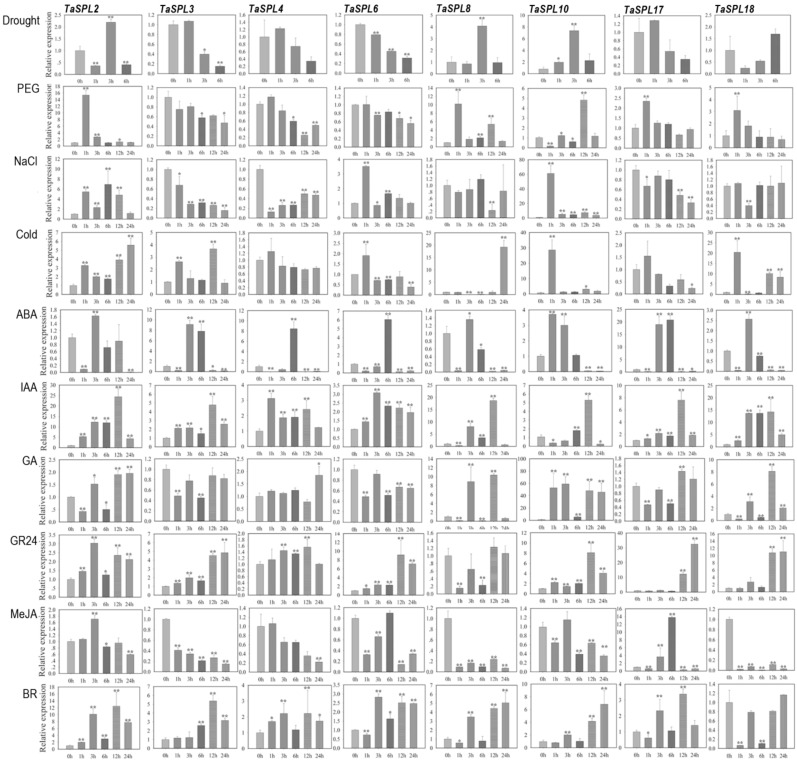
Spatial–temporal expression preference of *TaSPL*s and their responses to abiotic and phytohormone treatments, as determined by qPCR. Temporal expression profiles of *TaSPL2*, *TaSPL3*, *TaSPL4*, *TaSPL6*, *TaSPL8*, *TaSPL10*, *TaSPL17,* and *TaSPL18* at 0, 1, 3, 6, 12, and 24 h after the treatments of abiotic stresses or phytohormones. Significant differences in expression levels were determined by comparing each treatment at each time point with that at 0 h for each gene per treatment using Student’s *t*-test (*p* < 0.05). * and ** indicates significant difference at *p* < 0.05 and *p* < 0.01, respectively, in gene expression levels compared to that at 0 h for each treatment.

Our qPCR analysis demonstrated that *TaSPL* genes responded quite differently to the same treatment, and a *TaSPL* gene also showed distinct responses, in terms of expression, to the stresses and phytohormone treatments (Figure 4). The expression of *TaSPL2* was significantly upregulated after 1 h of drought treatment. *TaSPL2* was also upregulated after 6 h of combined drought and heat stresses (Figure 4). Under drought stress, eight *TaSPL* genes (*TaSPL2/3/4/6/8/10/17/18*) were up- or downregulated, with different stress-response patterns. *TaSPL6* was downregulated throughout the whole process of drought stress, while *TaSPL10* was dramatically upregulated (by over seven-fold) after 3 h of treatment. After PEG treatment, the expression of *TaSPL2/4/6/8/18* peaked at 1 h, *TaSPL10* peaked at 12 h, while *TaSPL3* was downregulated. Different from PEG treatment, NaCl treatment induced the expression of *TaSPL2/6/10* but repressed the expression of *TaSPL3/4/17*. Under cold treatment, *TaSPL6/10* responded rapidly at 1 h, while *TaSPL2/3/18* had two upregulation peaks in response.

Previous studies have shown that *SPL* genes respond to drought [67], heat [68], auxin (IAA), and brassinolide signal pathways [46], and biotic and abiotic stresses [34,67]. For the present study, we investigated the expression of *TaSPL*s expression under several phytohormone treatments, including ABA, IAA, GA, GR24, MeJA, and BR (Figure 4). Except for GA and MeJA, the remaining four phytohormones induced the expression of the analyzed *TaSPL*s, with IAA exhibiting the highest upregulation of *TaSPL*s (ranging from ~3-fold to over 25-fold upregulation). IAA mediated strong upregulation of *TaSPL2/8/10/17* at 12 h, while *TaSPL4/6/18* responded to IAA treatment earlier: at 1 to 3 h. For ABA treatment, all of the eight *TaSPL* genes showed a similar up-and-down expression pattern, with the majority of *TaSPL* expression induced in 3–6 h. The expression patterns of *TaSPL*s in response to GA were complex. *TaSPL3/6* were downregulated by GA; *TaSPL2/17* were first downregulated at early stages and then upregulated by GA at late stages; while *TaSPL8/10/18* expression was significantly induced by GA treatment. Unlike the complex responses to GA treatment, a few *TaSPL* genes (*TaSPL3/6/10/17/18*) were upregulated at late stages (12 to 24 h) under GR24 treatment. As the only *TaSPL*-repressing phytohormone, MeJA induced downregulation of several *TaSPL*s, including *TaSPL2/4/6/8/10/18*. *TaSPL17* was the only *TaSPL* gene significantly upregulated after MeJA treatment. For the BR treatment, several *TaSPL* genes (i.e., *TaSPL2/4/6/8/10/17*) exhibited two upregulation peaks—one peak at 3 h and the other at 12 h—while *TaSPL18* was downregulated by BR. These results suggest that the functions of some *TaSPL*s are (directly or indirectly) related to abiotic stress tolerance mechanisms and other biological processes, such as phytohormone biosynthesis, degradation, and signaling, thus regulating the plant growth and development of wheat. 

### 2.3. TaSPL3 Encodes a SPL Transcription Factor Highly Expressed in Young Spikes

When analyzing the expression preference between *OsSPL*s and their corresponding orthologous *SPL*s in wheat, the ubiquitously expressed *OsSPL*s and *TaSPL*s have been suggested as sharing conserved and significant roles during the development of rice and wheat, respectively. In order to prove the concept that a *TaSPL* gene may have largely conserved or overlapping functions with its *OsSPL* ortholog, if both the *OsSPL* and *TaSPL* exhibit similar expression patterns, we performed experiments to study the functions of *TaSPL3*. The expression preference of *SPL3* in developing spikes and stems in both rice and wheat indicates that SPL3 could play significant and conserved biological roles in the development of spikes and/or stems.

**Figure 5:**
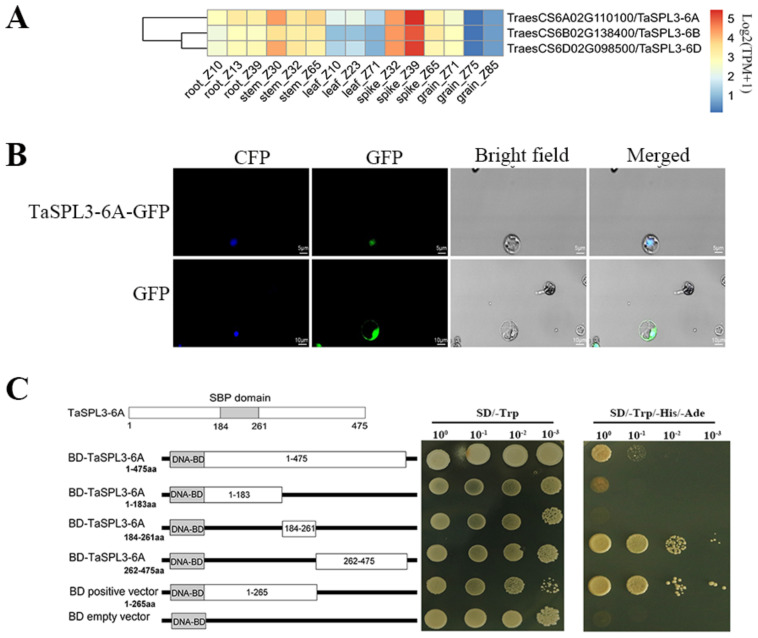
Characterization of the *TaSPL3* expression patterns (**A**), sub-cellular localization (**B**), and transactivation ability (**C**). (**A**) RNA-seq analysis demonstrates that all of the three TaSPL3 homeologous genes are highly expressed in spike tissues, followed by stem and developing grain tissues; (**B**) a sub-cellular localization assay was performed in isolated rice protoplasts with transient expression of *TaSPL3-6A* gene fused with *GFP*. CFP is a marker plasmid with nuclear localization as positive control; and (**C**) transcription activity analysis of TaSPL3-6A. In the left panel, the schematic diagram shows that the TaSPL3-6A protein is separated into three parts by the SBP domain (from 184 to 261 amino acid residues). In addition, the left panel shows a series of TaSPL3-6A full-length and truncated proteins that were constructed on the pGBKT7 vector, respectively, to identify the region of TaSPL3 with transactivation ability. In the right panel, the yeast colonies with four different gradients, 10^−1^, 10^−2^, 10^−3^, 10^−4^, were plated on the screening medium SD/−Trp and SD/−Trp/−His/−Ade, respectively. The empty pGBKT7 vector and positive vector were used as the negative and positive controls, respectively. SD: Synthetic dropout medium; SD/−Trp: Trp-defective SD; SD/−Trp/−Ade/−His: Trp-, Ade-, and His-defective SD.

*TaSPL3* consists of three highly conserved homeologous copies, *TaSPL3-A* (TraesCS6A02G1101001.2), *TaSPL3-B* (TraesCS6B02G138400.1), and *TaSPL3-D* (TraesCS6D02G098500.1), with over 96% identity for both the nucleotide and amino acid sequences. According to the public RNA-seq results of different tissues across wheat developmental stages, *TaSPL3* triads exhibit particularly high expression in developing spikes, moderate expression in stems and roots, low expression in leaves, and almost no expression in seeds (Figure 5A). *TaSPL3-A* has relatively higher expression, compared with *TaSPL3-B* and *-D*. Similarly, qPCR analysis of *TaSPL3* validated that it has the highest expression in young spikes, with moderate expression in other green tissues at different stages (Figure 3A and Figure S6).

To substantiate the functional study of *TaSPL3*, we investigated the sub-cellular localization of TaSPL3 (Figure 5B). TaSPL3-A-GFP fused protein was specifically localized in the nucleus, matching its role as a transcription factor. Furthermore, we performed a transactivation assay to determine the self-activation activity of TaSPL3 (Figure 5C). TaSPL3-A was truncated into three parts: N-terminal (1–183 amino acids), the SBP domain (middle 184–261 amino acids), and C-terminal (262–475 amino acids) (for primers, see Table S1). Our results showed that the N-terminal and SBP domain do not possess transactivation activity, while the C-terminal and the full-length TaSPL3-A can activate transcription. These results indicate that TaSPL3 is a nucleus-localized transcription activator.

### 2.4. Ectopic Expression of TaSPL3-A in Transgenic Rice Has Pleiotropic Effects on Plant Growth and Development

#### 2.4.1. Ectopic Expression of TaSPL3-A in Transgenic Rice Promotes Heading

To investigate the function of *TaSPL3* in rice, we generated transgenic lines of rice ubiquitously expressing *TaSPL3-A*, fused with the 3 × myc tag to facilitate protein detection (Figure S8). Six transgenic lines of *TaSPL3-A* (hereafter referred to as the TaSPL3-OE lines) were obtained, together with one line transforming the empty vector (the vector control line, VC) as a negative control. PCR results confirmed that the six TaSPL3-OE lines were *TaSPL3*-positive, with various expression levels of *TaSPL3*, as determined by qPCR (Figure S9). To investigate the phenotypic effects of ectopically expressed *TaSPL3* in rice, the transgenic lines and controls (VC and wild-type *cv*. Nipponbare) of the T_2_ generation were grown in the experimental field (Wuhan, China) using a complete random block design. Meanwhile, another batch of transgenic and control lines were grown in pots and placed beside the experimental field for ease of observation.

**Figure 6:**
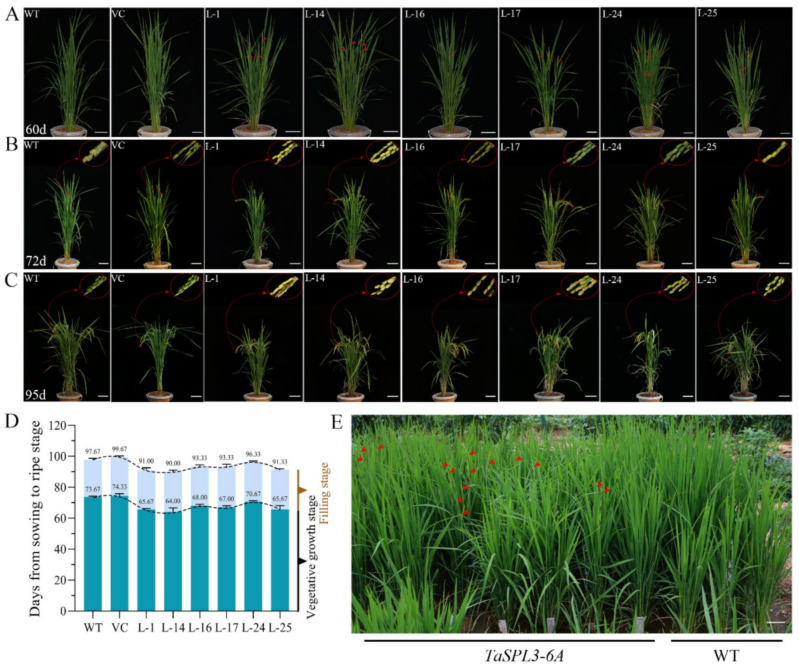
Ectopic expression of *TaSPL3* in transgenic rice exhibited early heading phenotype in both greenhouse (**A**–**D**) and fields (**E**). TaSPL3-OE lines headed earlier than the WT plants in 60 days (**A**), with the TaSPL3-OE panicles becoming mature earlier, as seen at 72 (**B**) and 95 (**C**) days of development. Statistical analysis revealed that the vegetative phase, rather than the reproductive phase, was shorter in the TaSPL3-OE lines than in the WT (**D**). The early heading phenotype was observed for the TaSPL3-OE lines in the T_2_ generation in experimental fields. Heading panicles are indicated by red arrows (**E**).

The ectopic expression of *TaSPL3* in rice promotes heading. The period from sowing to mature ranged from ~91 to ~96 days for the six TaSPL3-OE lines, while the same period for the control lines was 97 to 99 days (Figure 6). We found that the TaSPL3-OE lines started to head as early as ~60 days after sowing, while the control lines headed at ~72 days after sowing. Statistical analysis showed that the ectopic expression of *TaSPL3* led to 6–7 days earlier heading.

#### 2.4.2. Ectopic Expression of TaSPL3-A in Transgenic Rice Influences Leaf and Stem Development

**Figure 7:**
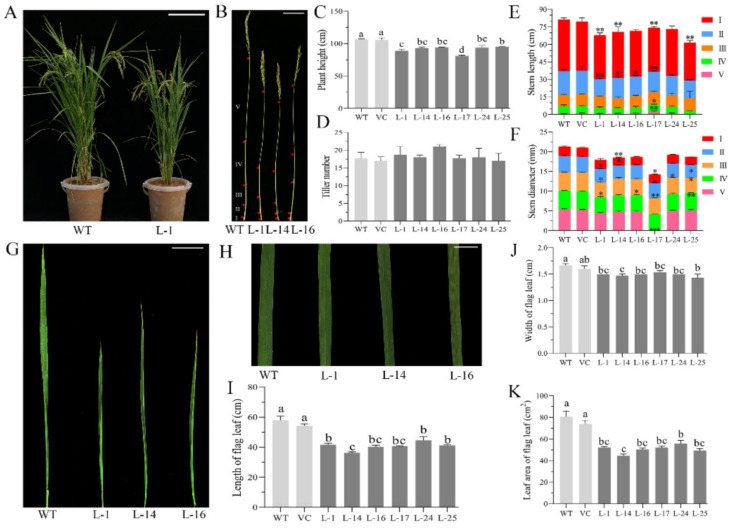
Ectopic expression of *TaSPL3* affected stem and leaf development in transgenic rice plants: (**A**) Morphological differences were observed between TaSPL3-OE transgenic lines (here, line L-1 as a representative) and wild-type rice (WT). Plant height and flag leaf size of TaSPL3-OE transgenic rice plants. (**B**–**F**) Detailed comparison of stem length and the number of internodes between TaSPL3-OE transgenic lines and WT shows that both the internode lengths (**E**) and stem diameters (**F**) in transgenic rice plants were significantly shorter than those in WT, leading to shorter plant heights of the transgenic plants (**C**). Tiller numbers of the TaSPL3-OE transgenic lines did not differ from WT (**D**). The length (**E**) and diameter (**F**) of each internode (I to V) were compared. Morphological comparison and detailed measurements showed that both the flag leaf length (**G**, **I**) and flag leaf width (**H**, **J**) in TaSPL3-OE transgenic lines were significantly lower than those in WT, leading to decreased flag leaf areas (**K**) in TaSPL3-OE transgenic lines. Statistical differences of the traits were determined using Tukey’s test, with mean values marked with different letters differing significantly (*p* < 0.05) among the lines. For figure (**E**) and **F**, * and ** indicates significant differences in stem lengths or diamters at *p* < 0.05 and *p* < 0.01, respectively, compared to those of the wildtype plants (determined by Student’s *t*-test).

Ectopic expression of *TaSPL3* also led to smaller plants with shorter stems and narrower leaves, in comparison to the control lines (Figure 7). The plant height of the control lines (WT and VC) was about 102.99–107.27 cm, while that of the TaSPL3-OE lines ranged from 79.9–95.5 cm (Figure 7A–D; Table S4). To identify detailed reasons for the shorter plant stature of TaSPL3-OE lines, we compared the length and diameter for each internode between the transgenic and control lines. Our results showed that, compared with the control lines, the TaSPL3-OE lines had shorter internode I and II, with some of the internode diameters also being smaller (Figure 7E,F); however, there was no obvious difference in tiller number between the transgenic and control lines, as shown in Figure 7D. In addition, we found that the TaSPL3-OE lines also affected the length, width, and area of the flag leaf (Figure 7G–K). These results clearly indicated that *TaSPL3* affects vegetative growth, but it remains to be investigated whether the decreased flag leaf and internode sizes were indirectly due to early heading, or due to ectopically expressed *TaSPL3* influencing the development of leaves and internodes.

#### 2.4.3. Ectopic Expression of TaSPL3-A in Transgenic Rice Affects Panicle Structures

**Figure 8:**
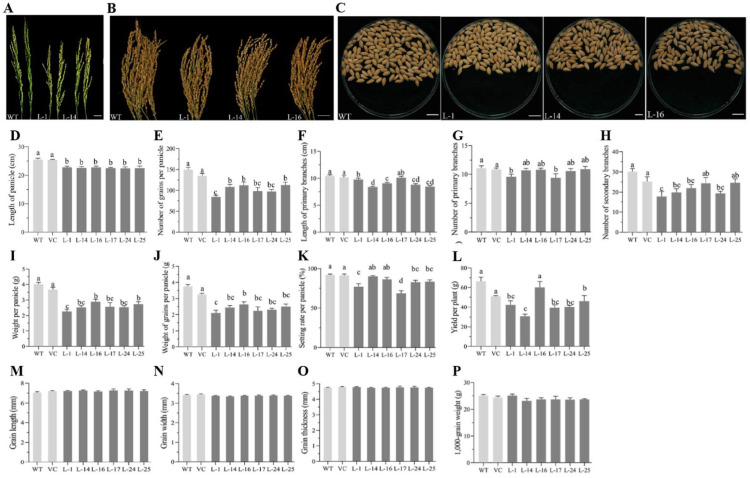
Ectopic expression of *TaSPL3* significantly affected panicle and yield traits in transgenic rice lines. Comparison of panicle morphology at immature (**A**) and mature stages (**B**), as well as grains per panicle (**C**) between TaSPL3-OE transgenic lines and wild-type plants, showed that ectopic expression of *TaSPL3* led to shorter panicles with fewer secondary branches. (**D**–**P**) Detailed analyses of panicle traits and grain traits demonstrated that ectopic expression of *TaSPL3* affected some panicle traits, but not grain traits (**M**–**O**) and thousand kernel weight (**P**). The analyzed panicle traits included length of primary branches (**F**), number of primary branches (**G**), number of secondary branches (**H**), weight per panicle (**I**), weight of grains per panicle (**J**), number of vacant grains per panicle (**K**), and yield per plant (**L**). The bar plots show mean values and standard error of the mean (S.E.M.), with mean values marked by different letters differing significantly (*p* < 0.05) among the lines. Statistical differences were determined by Tukey’s test.

As young panicles are the tissue where *SPL3* is primarily expressed, we investigated the phenotypic effects of *TaSPL3* overexpression in rice. Ectopic overexpression of *TaSPL3* affected the panicle size and structure, but did not influence grain-related traits (Figure 8). Expression of *TaSPL3* in rice led to smaller panicles (Figure 8A–C), primarily due to shorter primary branches and a decreased number of secondary branches and, hence, a reduced number of grains per panicle (Figure 8D–J). Slight differences in the number of primary branches and setting rate were also observed for the TaSPL3-OE lines, when compared to the control lines (Figure 8I–K). As for the grain-related traits, grain length, width, thickness, and thousand kernel weight were not affected by *TaSPL3* expression (Figure 8M–P). Collectively, *TaSPL3* expression resulted in lower yield, when compared to the non-transgenic *cv*. Nipponbare.

## 3. Discussion

### 3.1. Divergence between OsSPLs and TaSPLs

Due to the functional importance of *SPL*s in plant growth and development, the *TaSPL* genes have previously been identified in a genome-wide manner [41,42,43,44]. Consistent with the previous studies, we found 56 *TaSPL* genes present in the wheat reference genome of CS. Interestingly, *TaSPL*s have experienced different evolutionary trajectories, compared to the *OsSPL*s. The evolutionary differences of the *SPL* family are reflected in the following three aspects:

First, the paleoduplicated *SPL* gene pairs were not retained in common wheat (Figure 1). The previous study has shown that the most recent whole-genome duplication in rice has led to five duplicated pairs of *OsSPL* genes (pair 1: *OsSPL3*/*12*; pair 2: *OsSPL4*/*11*; pair 3: *OsSPL5*/*10*; pair 4: *OsSPL14*/*17*; and pair 5: *OsSPL16*/*18*). All five *OsSPL* pairs are retained and exhibit partially redundant functions and evidence for neofunctionalization [5,10,13,14,31,33,37,50,69]. For example, *OsSPL3* knockout altered the heading date, whereas *OsSPL12* knockout did not; *OsSPL4* knockout altered the heading date, whereas *OsSPL11* knockout did not; *OsSPL5* knockout changed the tiller number, whereas *OsSPL10* knockout did not [31]. In wheat, we did not find *TaSPL11* and *TaSPL12*, suggesting that their functions may have been replaced by other *SPL* members.

Second, *TaSPL*s differ from *OsSPL*s in terms of gene duplication patterns. During the allohexaploidization process of the wheat genome, the number of *TaSPL* genes was not only tripled by polyploidization, but also increased by tandem duplications, such as the case of *TaSPL10a*, *10b*, *10c,* and *10d* (Figure 1). A preliminary analysis regarding *TaSPL* gene duplication has been reported [44]; however, attempts have not previously been made to reconstruct the evolutionary relationships between *OsSPL* and *TaSPL*. Evolutionary comparison between the wheat and rice genomes clearly shows the expansion of the wheat genome by multiple mechanisms, including whole-genome duplications (WGD), tandem duplications and segmental duplications [70,71,72]. In rice, the WGD which occurred 70 to 90 million years ago (MYA) strongly impacted the rice genome and served as one of the major driving forces of gene duplication and divergence [73,74,75]. The WGD in the rice genome also led to the duplication of *OsSPLs* and drove subfunctionalization within the *OsSPL* pairs [31]. Unlike the rice genome, the wheat genome is characterized by a huge proportion of transposable elements (TEs, about 80% to 90%), polyploidization and a recent burst of gene duplications (RBGD) [72,76,77]. Indeed, our work demonstrates that the polyploidization and tandem duplications represent the major evolutionary force to drive the expansion of *TaSPL*s, differing from the case of *OsSPL*s.

Third, some *TaSPL* genes adopted distinct expression patterns, compared with those of *OsSPL*s. For example, *TaSPL5*, *7*, *10*, *14*, and *17* exhibited tissue-specific expression profiles, while other *SPL*s (e.g., *TaSPL2*, *13, 16*, and *18*) were preferentially expressed in some tissues and stages, but barely expressed in some other tissues, such as leaf sheaths, roots, stems, and seeds (see Figure 2A). Unlike *TaSPL*s, *OsSPL*s generally show tissue or organ expression preferences, but do not exhibit very specialized expression patterns (Figure 3 and Figure S7) [23]. In rice, the previous study has shown that the expression patterns of *OsSPL*s are associated with their functions. For example, the duplicated pair of *OsSPL3* and *OsSPL12* are expressed in leaves and panicles, with *OsSPL3* expressed higher in leaves and *OsSPL12* expressed higher in panicles, which partly explains the phenotypic effects of *OsSPL3* or *OsSPL12* knockout lines [31]. In addition, altered expression levels or expression patterns between the homeologous copies of a certain *TaSPL* further impacts their functions. For instance, we observed that *TaSPL8-D* was specifically and strongly expressed in leaf ligules followed by *TaSPL8-B* and *TaSPL8-A*. Indeed, the CRISPR-mediated knockout lines of *TaSPL8* homeologous copies, respectively, have proved that *TaSPL8-D* plays a determinant role in leaf-ligule development, while *TaSPL8-B* has only a moderate phenotypic effect, consistent with its expression level [46].

### 3.2. Toward Linking the Functions between OsSPLs and TaSPLs

Rice is the model species for gene function studies in monocot plants. It has several advantages when being used to facilitate the functional study of *TaSPL* genes: (1) rice has a similar plant architecture to wheat; (2) the orthologous relationships between *TaSPL*s and *OsSPL*s are studied and reported herein; (3) rice has extensive gene expression data sets and several well-established expression databases [78,79,80]; (4) extensive functional studies have been reported, using forward genetics, mutants, transgenic, or genome-editing approaches to characterize *OsSPL* members (as summarized in Table S5); and (5) rice is a monocot species that can be easily transformed and genome-edited with high efficiency, making it a prime model system for heterologously investigating the effects of *TaSPL*.

Unlike the monocot model specie rice, the genetic transformation of wheat has been established for decades [81,82,83,84], while creating genome-edited plants has only recently become possible in wheat [85,86]. The transformation efficiency in wheat has been improved recently, by optimizing the *Agrobacterium*-mediated transformation system [87]. Nevertheless, the transformation efficiency in wheat is not comparable to that in rice, nor have improved wheat transformation systems become widely used as a routine technique yet. Therefore, comparisons between *OsSPL*s and *TaSPL*s, in the aspects of gene orthology, expression patterns, and functions may indicate the functions of *TaSPL* genes, thus helping to prioritize the *TaSPL* genes for detailed genetic and functional studies. To this end, we collected the known functions of *OsSPL* genes based on previous reports [5,6,11,13,14,30,31,32,33,34,35,36,37,44,69] (Table S5). Consistent with the observations that most *OsSPL* genes are widely expressed in several organs—including leaves, stems, inflorescences, and seeds—with expression preferences in certain tissues and stages (Figure S7), several published investigations have revealed the pleiotropic functions of OsSPLs, and the complex functional redundancy between the OsSPL members (summarized in Table S5). For example, transgenic and mutant studies have unveiled that many *OsSPL*s (i.e., *SPL3*, *4*, *7*, *9*, *10*, *13*, *14*, *16*, *17,* and *18*) have impacts on plant height, flowering time, panicle structure, and grain development [5,31,33,34,36,37,50]. Similarly, the paleoduplicated pairs of *OsSPL* genes exhibit overlapped functions with sub-functionalizations in some traits, which are likely contributed to by the differentiated expression between pairs of *SPL* genes [31].

Owing to the important relatedness between the expression patterns and gene functions as demonstrated in *OsSPL*s, the expression patterns between *OsSPL*s and *TaSPL*s have been compared in the present study (Figure 2, Figure 3, and Figure S7). In such comparative analyses, we acknowledge that several limitations hamper the direct comparison of expression levels between rice and wheat. The widely used expression data sets in rice have been generated by microarray (Figure S7), while wheat expression has been profiled more recently by using RNA-seq (Figure 2) [78,79,80,88,89,90]. Therefore, we compared the organs where *OsSPL* and *TaSPL* genes were preferentially or highly expressed (Figure 3), utilizing the concept that, if a gene shows particularly high expression in a certain tissue, it likely exerts an important function in that tissue. Following this concept, we discovered that both *OsSPL3* and *TaSPL3* shared a similar expression pattern, being highly expressed in leaves, roots, stems, inflorescences, and flower organs (Figure 2, Figure 3 and Figure 5A). As a proof of concept, we sought to validate the function of *TaSPL3* in transgenic rice. Indeed, ectopic expression of *TaSPL3* in rice affected flowering time, plant height, flag leaf development, and panicle structures, but did not alter tiller number or grain size (Figure 6, Figure 7 and Figure 8). Similarly, a previous research work has demonstrated that the *OsSPL3* knockout by using CRISPR-mediated genome editing also modified plant height, flowering time, and panicle-related traits, supporting the conserved functions between *OsSPL3* and *TaSPL3* [31]. Other lines of evidence also support the concept that where a *SPL* is highly expressed affects its functions. For example, *TaSPL8* is specifically expressed at leaf ligules (Figure 2A). The knockout lines of *TaSPL8-A*, *-B*, and *-D*, respectively, proved that *TaSPL8* controls leaf ligule development, with *TaSPL8-D* having the highest expression levels and phenotypic contribution [46]. By contrast, *OsSPL8* are expressed in leaves, panicles and developing seeds (Figure S7). Matching with the expression pattern, functional characterization of *OsSPL8* proved that it not only controls leaf ligule development, but also affects plant height, panicle size, and grain length [31]. In rice, several forward genetic studies have demonstrated that the natural alleles of *OsSPL*s with elevated and/or ectopic expression confer agronomically desirable traits in different accessions of rice or wild rice [5,10,11,13,36,91,92]. Natural variation in the promoter region leads to decreased expression of *OsSPL10* and regulates trichome development in rice cultivars [91]. Natural variation of *OsSPL14* in rice causes its deregulation by miR156 and higher expression levels in the developing tissues, led to the *Ideal Plant Architecture* (*IPA*) phenotypes with less tillers, bigger panicles and bigger grains [10]. Because the TaSPL-OsSPL comparison described here highlighted gene divergence and differences in expression patterns, it is suggested that *TaSPL*s could provide a novel genetic resource to modify the growth, development, and yield in cereal crops.

Another limitation for the comparison between *OsSPL*s and *TaSPL*s lies in the miRNAs that regulates *SPL* genes. In rice, miR156 and miR529 are known to target some *OsSPL* [10,36,93], while the expression profiling of miR156 and miR529 have not been reported in wheat. Only a few studies in wheat have annotated and profiled miRNAs [92,94,95]. In particular, molecular characterization of the miR529 family has not been carried out in wheat. Integrated multi-omics analysis combining both expression data sets of *TaSPL* genes and their regulatory miRNAs (miR156 and miR529) are expected to be indispensable and useful in gaining a thorough understanding of the pleiotropic functions of *TaSPLs* in the future [96].

## 4. Materials and Methods

### 4.1. Genome-Wide Identification of TaSPLs and Phylogenetic Analysis

The annotated protein-coding genes from the reference genome of the wheat cultivar Chinese Spring (CS) were used to search for *SPL* genes with HMMER (E-value < 0.01), and the SBP domain was obtained from SMART and InterPro databases [97,98]. Eighteen *AetSPL*s, ten *TuSPL*s, sixteen *BdSPLs*, and nineteen *OsSPL*s from *Aegilops tauschii* (v4.0 genome), *Triticum urartu* (v1), *Brachypodium distachyon* (v3.0), and *Oryza sativa* (*IRGSP* v1.0), respectively, which have been reported elsewhere, were used in the present study for phylogenetic analysis [23,43,44]. The genome information of these monocot species is available from EnsemblPlant (http://plants.ensembl.org/) (accessed on 6 December 2021). Information about the *TaSPL*s is provided in Table S1.

Phylogenetic analysis of the *SPL* gene family was performed using the maximum-likelihood method with 1000 bootstraps, using MEGA X for *TaSPL*s, *AetSPL*s, *TuSPL*s, *BdSPL*s, *OsSPL*s, and *AtSPL*s [99]. The full-length protein sequences of SPLs were used.

### 4.2. Analyses of Sequence Alignment, Protein Domains, and Conserved Motifs

The protein sequences of OsSPLs and the 56 TaSPL identified in the present study were aligned using ClustalW 2.0, in order to determine the SBP domain [100]. The genomic and cDNA sequences of the 56 *TaSPL* genes were retrieved from the wheat reference genome, and the exon–intron structures of *TaSPL*s were analyzed using TBtools [101]. Protein motifs conserved in TaSPLs were identified using MEME [102].

### 4.3. Syntenic Analysis of SPL Genes between Wheat and Rice

The chromosomal locations of the 56 *TaSPL* genes were visualized using TBtools (Figure 1B). Syntenic relationships between *OsSPL*s and *TaSPL*s were established using TriGeneTribe and MCScanX (Figure S2) [103]. Based on this result, the nomenclature of *TaSPL* genes was compared, based on several previous studies on the *TaSPL* family, and adjusted in the present study to reflect the *SPL* syntelogous connections between wheat and rice (Table S1) [42,43,44].

### 4.4. Plant Materials

The wheat cultivar Chinese Spring was planted in the experimental field of Huazhong University of Science and Technology (Wuhan, China) for *TaSPL* gene cloning and expression analysis. The rice (*Oryza sativa* L. japonica) cultivar “Nipponbare” plants grown in greenhouse were used for rice protoplast preparation and transformation. To study the phenotypic effects of TaSPL3, transgenic lines of rice expressing *TaSPL3-6A* were generated (see Method Section 4.9). The transgenic lines of rice with ectopic *TaSPL3-6A* expression or with the *TaSPL3pro*:*uidA* expression cassette were also grown under the field conditions for molecular and phenotypic studies.

### 4.5. Gene Expression Analysis

Two wheat expression databases—WheatOmics and expVIP—were used to retrieve the gene expression profiles of *TaSPL* genes [61,89]. To examine the expression patterns of *TaSPL* across different tissues and stages during development, three RNA-seq data sets were used: one from wheat cultivar Azhurnaya [62] (the data set “BCS” in Figure 2A), one from the different tissues of developing grain of *cv*. Chinese Spring [90] (the data set “Dev_Grain” in Figure 2A), and one from the immature inflorescences of cultivar Kenong9204 [88] (the data set “Spikes” in Figure 2A). All of the gene expression data were presented in transcript per million (TPM), and then converted to z-scores for heatmap visualization using the “pheatmap” function from the R package “COMPASS” (http://www.bioconductor.org/packages/devel/bioc/html/COMPASS.html) (accessed on 22 November 2021). When identifying sub-genome expression biases, the largest data set in wheat (namely, BCS) was used, in order to avoid potential batch effects between the RNA-seq data sets, with the statistical differences of expression between sub-genomes calculated by two-tailed Student’s *t*-test (Figure 2B). RNA-seq data and qPCR data from previous studies were used to analyze the expression patterns of *TaSPL*s in response to abiotic stresses (primers in Table S2; previous results summarized in Table S3) [47,49,83,84].

Two rice expression databases—RiceXpro and the rice expression database (RED)—were used to retrieve the gene expression profiles of *OsSPL* genes [79,80]. As several *OsSPL*s are involved in grain development, four data sets in RiceXPro were chosen, including RXP_0001 (spatial–temporal expression of various tissues throughout entire growth period) [78], RXP_0010 (gene expression profile during reproductive organ development), RXP_0011 (gene expression in grains at early developmental stages), and RXP_0012 (gene expression in embryos and endosperms at ripening stages). These four RiceXPro data sets provided microarray-based gene expression profiles with similar developmental tissues and stages to the *TaSPL* expression. The expression analysis of rice microarray data sets has been described elsewhere [78]. To facilitate the data comparison and heatmap visualization across several data sets, the microarray-based expression values were z-score transformed and scaled within the range from −1 to 1 (Figure S7A). The RNA-seq based *OsSPL* expression data from the RED were used to validate the *OsSPL* preferentially expressed tissues (Figure S7B). As many RNA-seq data sets collected in the RED have placed emphasis on the expression responses to nutritional elements, abiotic, and biotic stresses, the *OsSPL* expression obtained from the RED were not used for comparison with that of *TaSPL*s. Due to technical difficulties in direct comparison between microarray- and RNA-seq-based expression, we grouped the *TaSPL* expression data (RNA-seq based) and *OsSPL* expression data (microarray-based) first by organs and then by tissues and stages, and generally compared preferentially expressed organs and the relative expression abundance at the organ level (Figure 3).

### 4.6. qPCR-Based Expression Profiling of TaSPLs

The expression profiles of *TaSPL* genes were analyzed using quantitative PCR (qPCR) in different tissues across the developmental stages and under several abiotic stresses or phytohormone treatments.

Plant samples were collected at various vegetative and reproductive stages, according to the scales of Zadoks and Tottman for cereals [104,105]. The samples included coleoptiles and radicles (germination stage, Z00–Z09); roots, culm bases, leaves, and leaf sheaths (seedling stage, Z10–Z19); tiller base, leaf sheath, axillary buds, and pulvinus (tillering stage, Z20–Z29); nodes, internodes, and flag leaves (stem elongation stage, Z30–Z39); ligules and auricles (heading stage, Z40–Z49); stamens, pistils, and young spikes of varying lengths (YS4, YS5, YS6, YS8, YS9, YS12; YS means young spikes and each Arabic numeral represents the number of centimeters; flowering stage, Z50–Z69); and developing seeds (DAP3, DAP5, DAP8, DAP10, DAP15, DAP20, DAP25, DAP28; DAP means days after pollination and each Arabic numeral represents the number of days; Endosperm development, Z70–Z89). The collected samples were quickly frozen with liquid nitrogen and stored at −80 °C. To examine the responses of *TaSPL* genes to various stresses, previously reported RNA-seq expression results were used (see Method Section 4.5; Table S3).

Quantitative PCR experiments were performed to validate the expression patterns of selected *TaSPL* genes (*TaSPL2*/*3*/*4*/*6*/*8*/*10*/*17*/*18*) which were differentially expressed in the RNA-seq analysis (Figure 4, Table S3). To do this, two-week-old seedlings of wheat cv. CS were treated with abiotic stress—such as drought (dehydration), PEG 6000 (20% *w*/*w*), NaCl (200 mM), or cold (4 °C)—or with phytohormone addition—such as abscisic acid (ABA, 100 μM), indole-3-acetic acid (IAA, 50 μM), gibberellic acid (GA, 50 μM), GR24 (a synthetic strigolactone, 6 μM), methyl jasmonic acid (MeJA, 100 μM), or brassinosteroid (BR, 50 μM)—for 1, 3, 6, 12, or 24 h to examine the stress tolerance-related or phytohormone-mediated regulation of *TaSPL* genes. Seedlings treated with distilled water were used as a control.

Total RNA was extracted by using an RNA Extraction Kit (Zomanbio, Beijing, China) and reversely transcribed into cDNA for qRT-PCR as described previously [106]. qRT-PCR was carried out using SYBR Green Master Mix on a CFX96 real-time System (Bio-RAD, Hercules, CA, USA), with three biological replicates for each sample or treatment. *TaActin* (TraesCS1B02G283900) was used as the internal reference gene for qPCR. The qPCR program included pre-denaturation at 95 °C for 10 min and 40 cycles of denaturation at 95 °C for 10 s, annealing at 60 °C for 30 s, and extension at 72 °C for 1 min. The primers used for qPCR are provided in Table S2.

### 4.7. Gene Cloning of TaSPL3-6A and the Sub-Cellular Localization of TaSPL3-6A

To carry out the functional study, *TaSPL3-6A* was amplified from the cDNA of wheat young spikes with the following PCR program: pre-denaturation at 98 °C for 30 s and 35 cycles of denaturation at 98 °C for 10 s, annealing at 55–65 °C for 15 s, and extension at 72 °C for 1 min (primer sequences in Table S2). The *TaSPL3-6A* sequence was verified using Sanger sequencing at the AuGCT company (Beijing, China).

The coding region of *TaSPL3-6A* was fused with GFP ORF through XbaI/BamHI restriction sites to obtain the construct (namely, pSGN-TaSPL3-6A-GFP) for the sub-cellular localization experiment, in which the expression of *TaSPL3*-*GFP* was driven by the CaMV35S promoter. An empty vector, pSGN-GFP, was used as the negative control. Protoplasts of rice seedling leaves were prepared. Under an induction of 40% PEG-4000, empty or recombinant plasmids (pSGN-GFP or pSGN-TaSPL3-6A-GFP) were co-transformed into rice protoplasts with the marker plasmid (CFP). The transformed protoplasts were cultured at 28 °C for 8–10 h under dark conditions. A laser confocal microscope (FV1200, Olympus, Valley, PA, USA) was used to detect the sub-cellular localization of GFP proteins or TaSPL3-6A-GFP fusion proteins.

### 4.8. Transactivation Assay

The *TaSPL3-6A* coding region was truncated into three parts: The N-terminal (N), SBP domain (SBP), and C-terminal (C). Each of the three parts or the full-length of *TaSPL3-6A* was cloned into pGBKT7 plasmids through *Bam*HI/*Nco*I restriction sites, in order to obtain the recombinant constructs pGBKT7-TaSPL3-N, pGBKT7-TaSPL3-SBP, pGBKT7-TaSPL3-C, and pGBKT7-TaSPL3-6A-FL, respectively. According to the manufacturer’s protocol (Clontech, Foster City, CA, USA), the abovementioned recombinant constructs, as well as pGBKT7 and the positive control, were transformed into yeast strain AH109. The transformed yeast strains were diluted at different concentrations, then dotted onto SD/-Trp or SD/-Trp/-His/-Ade medium. After culturing for four days, the trans-activation activities of full-length *TaSPL3-6A* or its fragments were evaluated by the columns of transformed yeasts.

### 4.9. Generation of the Transgenic Rice Lines

To ectopically express *TaSPL3* in rice, the open reading frame (ORF) of *TaSPL3-6A* was fused with the 3-myc tag and then inserted into the *Agrobacterium* transformation vector pCAMBIA1304, with its expression driven by the CaMV 35S promoter. Rice transformation was performed using the *Agrobacterium* immersion method with strain *EHA105* and calluses induced from *cv*. Nipponbare immature embryos [107]. To determine transgenic positive events of rice, DNA was extracted from leaves of independent T_0_ plants, and specific PCR primers were designed to amplify the 383-bp fragment within the selection gene (*hygromycin B phosphotransferase* gene) or the 2161-bp fragment of the *TaSPL3-6A* gene. Quantitative PCR analysis was performed to evaluate *TaSPL3* expression levels in the leaves of transgenic rice plants in the T_0_ generation (all primer sequences provided in Table S1). Subsequently, the T_0_ plants were selected based on the aforementioned PCR and qPCR results, in order to propagate to T_1_ and T_2_ lines for phenotypic observation. Additionally, transgenic rice lines transformed with the empty vector pCAMBIA1304 (the vector control lines, VC) were also generated to serve as a negative control.

### 4.10. Phenotypic Analysis of TaSPL3-OE Transgenic Lines

The T_2_ lines of *TaSPL3-OE* and control lines (including both non-transformed *cv*. Nipponbare and VC) of rice were planted in a randomized block field experiment with three replicates at the experimental fields (Wuhan, China). In each plot, three rows of plants were grown with 25-cm row spaces. Within each row, about 15 individual plants were grown with 20-cm of plant spacing. Regular field managements, including irrigation, fertilization, and insect, disease, and weed control, were applied.

The growth periods for each line of rice, including seedling date, tiller date, stem elongation date, heading date, flowering time, and maturity time were observed. In each plot, 10 plants were chosen to measure various agronomic traits, including plant height, flag leaf sizes (leaf length, leaf width, and leaf area), tiller numbers, panicle length, numbers of primary and secondary branches per panicle, grain weight per panicle, grain numbers per panicle, seed-setting percentage per panicle, length, width, and thickness of grains, thousand-grain weight, and yield per plant. The length, width, and thickness of grains were measured using seed testing instrument (SC-G, Wanshen, Hangzhou, China). The significance of differences among means of agronomic traits was determined using Tukey’s honest significant difference test.

## 5. Conclusions

In the present study, we performed a comprehensive analysis of the *TaSPL* family, identified 56 *TaSPL* genes, and established the orthologous relationship between *TaSPL*s and *OsSPL*s. A detailed qRT-PCR analysis pinpointed several *TaSPLs*, *TaSPL2*/*6*/*8*/*10*, involved in the tolerance of different abiotic stresses. Our results highlighted the conservation and divergence between *TaSPL*s and *OsSPL*s. As a proof of the functional prediction from the dry lab data, we demonstrated that *TaSPL3* shares a conserved function with *OsSPL3* in regulating plant height, flowering time and panicle-related traits by using transgenic lines of rice. Importantly, our work leads to a clear take-home message that the combination of evolutionary and expression analyses can serve as an efficient approach to transfer the functional knowledge from the monocot model species rice to wheat, helping to gain better understanding of the functions of *TaSPL*s. The approach exemplified here may also be effective in functional characterization of agronomically important gene families in wheat, such as other transcription factors. 

## Data Availability

All of the data reported in this manuscript are provided in the Supplementary Files.

## Supplementary Materials

The following are available online at https://www.mdpi.com/article/10.3390/ijms23042099/s1.

## Abbreviations

ABA	Abscisic acid
BR	Brassinosteroid
CFP	Cyan fluorescent protein
CRISPR	Clustered regularly interspaced short palindromic repeats
DAP	Days after anthesis
GFP	Green fluorescent protein
GA	Gibberellin
HMM	Hidden Markov model
IPA	Ideal plant architectures
IAA	Auxin
IWGSC	International wheat genome sequencing consortium
MYA	Million years ago
MeJA	Methyl jasmonic acid
MCScanX	Multiple Collinearity Scan toolkit
MEGA	Simple modular architecture research tool
NLS	Nuclear localization signal
OE	Overexpression
ORF	Open reading frame
PEG	Polyethylene glycol
qPCR	Quantitative polymerase chain reaction
qRT-RCR	Real-time quantitative polymerase chain reaction
SBP	SQUAMOSA-PROMOTER BINDING PROTEIN
SPL	SQUAMOSA-PROMOTER BINDING PROTEIN-LIKE
SMART	Simple modular architecture research tool
SD	Synthetic dropout medium
SEB	Sub-genome expression bias
TPM	Transcripts per kilobase million
TF	Transcription factor
VC	Vector control
WT	Wild type
WGD	Whole-genome duplication
YS	Young spikes

## References

[1] Tu M., Li Y. (2020). Toward the genetic basis and multiple QTLs of kernel hardness in wheat. Plants.

[2] Sun Z., Su C., Yun J., Jiang Q., Wang L., Wang Y., Cao D., Zhao F., Zhao Q., Zhang M. (2019). Genetic improvement of the shoot architecture and yield in soya bean plants via the manipulation of *GmmiR156b*. Plant Biotechnol. J..

[3] Gou J., Fu C., Liu S., Tang C., Debnath S., Flanagan A., Ge Y., Tang Y., Jiang Q., Larson P.R. (2017). The *miR156*-*SPL4* module predominantly regulates aerial axillary bud formation and controls shoot architecture. New Phytol..

[4] Gallavotti A., Zhao Q., Kyozuka J., Meeley R.B., Ritter M.K., Doebley J.F., Pe M.E., Schmidt R.J. (2004). The role of barren stalk1 in the architecture of maize. Nature.

[5] Jiao Y., Wang Y., Xue D., Wang J., Yan M., Liu G., Dong G., Zeng D., Lu Z., Zhu X. (2010). Regulation of *OsSPL14* by *OsmiR156* defines ideal plant architecture in rice. Nat. Genet..

[6] Lu Z., Yu H., Xiong G., Wang J., Jiao Y., Liu G., Jing Y., Meng X., Hu X., Qian Q. (2013). Genome-wide binding analysis of the transcription activator ideal plant architecture1 reveals a complex network regulating rice plant architecture. Plant Cell.

[7] Xing S., Salinas M., Hohmann S., Berndtgen R., Huijser P. (2010). *MiR156*-targeted and nontargeted SBP-box transcription factors act in concert to secure male fertility in *Arabidopsis*. Plant Cell.

[8] Chuck G.S., Brown P.J., Meeley R., Hake S. (2014). Maize SBP-box transcription factors unbranched2 and unbranched3 affect yield traits by regulating the rate of lateral primordia initiation. Proc. Natl. Acad. Sci. USA.

[9] Gao R., Gruber M.Y., Amyot L., Hannoufa A. (2018). *SPL13* regulates shoot branching and flowering time in *Medicago sativa*. Plant Mol. Biol..

[10] Miura K., Ikeda M., Matsubara A., Song X.J., Ito M., Asano K., Matsuoka M., Kitano H., Ashikari M. (2010). *OsSPL14* promotes panicle branching and higher grain productivity in rice. Nat. Genet..

[11] Si L., Chen J., Huang X., Gong H., Luo J., Hou Q., Zhou T., Lu T., Zhu J., Shangguan Y. (2016). *OsSPL13* controls grain size in cultivated rice. Nat. Genet..

[12] Wang J.W., Schwab R., Czech B., Mica E., Weigel D. (2008). Dual effects of *miR156*-targeted *SPL* genes and *CYP78A5*/*KLUH* on plastochron length and organ size in *Arabidopsis thaliana*. Plant Cell.

[13] Wang S., Li S., Liu Q., Wu K., Zhang J., Wang S., Wang Y., Chen X., Zhang Y., Gao C. (2015). The *OsSPL16*-*GW7* regulatory module determines grain shape and simultaneously improves rice yield and grain quality. Nat. Genet..

[14] Wang S., Wu K., Yuan Q., Liu X., Liu Z., Lin X., Zeng R., Zhu H., Dong G., Qian Q. (2012). Control of grain size, shape and quality by *OsSPL16* in rice. Nat. Genet..

[15] Cardon G., Hohmann S., Klein J., Nettesheim K., Saedler H., Huijser P. (1999). Molecular characterisation of the *Arabidopsis SBP-box* genes. Gene.

[16] Yamasaki K., Kigawa T., Inoue M., Tateno M., Yamasaki T., Yabuki T., Aoki M., Seki E., Matsuda T., Nunokawa E. (2004). A novel zinc-binding motif revealed by solution structures of DNA-binding domains of *Arabidopsis* SBP-family transcription factors. J. Mol. Biol..

[17] Birkenbihl R.P., Jach G., Saedler H., Huijser P. (2005). Functional dissection of the plant-specific SBP-domain: Overlap of the DNA-binding and nuclear localization domains. J. Mol. Biol..

[18] Cardon G.H., Hohmann S., Nettesheim K., Saedler H., Huijser P. (1997). Functional analysis of the *Arabidopsis thaliana* SBP-box gene *SPL3*: A novel gene involved in the floral transition. Plant J..

[19] Klein J., Saedler H., Huijser P. (1996). A new family of DNA binding proteins includes putative transcriptional regulators of the *Antirrhinum majus* floral meristem identity gene *SQUAMOSA*. Mol. Gen. Genet..

[20] Hultquist J.F., Dorweiler J.E. (2008). Feminized tassels of maize *mop1* and *ts1* mutants exhibit altered levels of *miR156* and specific *SBP-box* genes. Planta.

[21] Li J., Hou H., Li X., Xiang J., Yin X., Gao H., Zheng Y., Bassett C.L., Wang X. (2013). Genome-wide identification and analysis of the SBP-box family genes in apple (*Malus × domestica* Borkh.). Plant Physiol. Biochem..

[22] Salinas M., Xing S., Hohmann S., Berndtgen R., Huijser P. (2012). Genomic organization, phylogenetic comparison and differential expression of the SBP-box family of transcription factors in tomato. Planta.

[23] Xie K., Wu C., Xiong L. (2006). Genomic organization, differential expression, and interaction of SQUAMOSA promoter-binding-like transcription factors and *microRNA156* in rice. Plant Physiol..

[24] Zhang L., Wu B., Zhao D., Li C., Shao F., Lu S. (2014). Genome-wide analysis and molecular dissection of the *SPL* gene family in *Salvia miltiorrhiza*. J. Integr. Plant Biol..

[25] Gandikota M., Birkenbihl R.P., Hohmann S., Cardon G.H., Saedler H., Huijser P. (2007). The *miRNA156/157* recognition element in the 3′UTR of the *Arabidopsis* SBP box gene *SPL3* prevents early flowering by translational inhibition in seedlings. Plant J..

[26] Wu G., Poethig R.S. (2006). Temporal regulation of shoot development in *Arabidopsis thaliana* by *miR156* and its target *SPL3*. Development.

[27] Zhang Y., Schwarz S., Saedler H., Huijser P. (2007). *SPL8*, a local regulator in a subset of gibberellin-mediated developmental processes in *Arabidopsis*. Plant Mol. Biol..

[28] Shikata M., Koyama T., Mitsuda N., Ohme-Takagi M. (2009). Arabidopsis SBP-box genes *SPL10*, *SPL11* and *SPL2* control morphological change in association with shoot maturation in the reproductive phase. Plant Cell Physiol..

[29] Stone J.M., Liang X., Nekl E.R., Stiers J.J. (2005). *Arabidopsis AtSPL14*, a plant-specific SBP-domain transcription factor, participates in plant development and sensitivity to fumonisin B1. Plant J..

[30] Dai Z., Wang J., Yang X., Lu H., Miao X., Shi Z. (2018). Modulation of plant architecture by the *miR156f*–*OsSPL7*–*OsGH3.8* pathway in rice. J. Exp. Bot..

[31] Jiang M., He Y., Chen X., Zhang X., Guo Y., Yang S., Huang J., Traw M.B. (2020). CRISPR-based assessment of genomic structure in the conserved *SQUAMOSA* promoter-binding-like gene clusters in rice. Plant J..

[32] Lee J., Park J.J., Kim S.L., Yim J., An G. (2007). Mutations in the rice liguleless gene result in a complete loss of the auricle, ligule, and laminar joint. Plant Mol. Biol..

[33] Shao Y., Zhou H.Z., Wu Y., Zhang H., Lin J., Jiang X., He Q., Zhu J., Li Y., Yu H. (2019). *OsSPL3*, an SBP-domain protein, regulates crown root development in rice. Plant Cell.

[34] Tang M., Zhou C., Meng L., Mao D., Peng C., Zhu Y., Huang D., Tan Z., Chen C., Liu C. (2016). Overexpression of *OsSPL9* enhances accumulation of Cu in rice grain and improves its digestibility and metabolism. J. Genet. Genom..

[35] Wang J., Zhou L., Shi H., Chern M., Yu H., Yi H., He M., Yin J., Zhu X., Li Y. (2018). A single transcription factor promotes both yield and immunity in rice. Science.

[36] Wang Q.L., Sun A.Z., Chen S.T., Chen L.S., Guo F.Q. (2018). *SPL6* represses signalling outputs of ER stress in control of panicle cell death in rice. Nat. Plants.

[37] Yuan H., Qin P., Hu L., Zhan S., Wang S., Gao P., Li J., Jin M., Xu Z., Gao Q. (2019). *OsSPL18* controls grain weight and grain number in rice. J. Genet. Genom..

[38] Brenchley R., Spannagl M., Pfeifer M., Barker G.L., D’Amore R., Allen A.M., McKenzie N., Kramer M., Kerhornou A., Bolser D. (2012). Analysis of the bread wheat genome using whole-genome shotgun sequencing. Nature.

[39] Dvorak J., Akhunov E.D. (2005). Tempos of gene locus deletions and duplications and their relationship to recombination rate during diploid and polyploid evolution in the *Aegilops-Triticum* alliance. Genetics.

[40] Zhang H.K., Zhu B., Qi B., Gou X.W., Dong Y.Z., Xu C.M., Zhang B.J., Huang W., Liu C., Wang X.T. (2014). Evolution of the BBAA component of bread wheat during its history at the allohexaploid level. Plant Cell.

[41] Guo F.Y., Lu Q.W., Cang J. (2021). Genome-wide identification and expression profiling of the *SPL* family genes in wheat. Botany.

[42] Li Y., Song Q., Zhang Y., Li Z., Guo J., Chen X., Zhang G. (2020). Genome-wide identification, characterization, and expression patterns analysis of the SBP-box gene family in wheat (*Triticum aestivum* L.). Sci. Rep..

[43] Song J.H., Ma D.F., Yin J.L., Yang L., He Y.Q., Zhu Z.W., Tong H.W., Chen L., Zhu G., Liu Y.K. (2019). Genome-wide characterization and expression profiling of squamosa promoter binding protein-like (SBP) transcription factors in wheat (*Triticum aestivum* L.). Agronomy.

[44] Zhu T., Liu Y., Ma L., Wang X., Zhang D., Han Y., Ding Q., Ma L. (2020). Genome-wide identification, phylogeny and expression analysis of the *SPL* gene family in wheat. BMC Plant Biol..

[45] Li L., Shi F., Wang Y., Yu X., Zhi J., Guan Y., Zhao H., Chang J., Chen M., Yang G. (2020). *TaSPL13* regulates inflorescence architecture and development in transgenic wheat (*Triticum aestivum* L.). Plant Sci..

[46] Liu K., Cao J., Yu K., Liu X., Gao Y., Chen Q., Zhang W., Peng H., Du J., Xin M. (2019). Wheat *TaSPL8* modulates leaf angle through auxin and Brassinosteroid Signaling. Plant Physiol..

[47] Zhang B., Xu W., Liu X., Mao X., Li A., Wang J., Chang X., Zhang X., Jing R. (2017). Functional conservation and divergence among homoeologs of *TaSPL20* and *TaSPL21*, two SBP-Box genes governing yield-related traits in hexaploid wheat. Plant Physiol..

[48] Cao R., Guo L., Ma M., Zhang W., Liu X., Zhao H. (2019). Identification and functional characterization of squamosa promoter binding protein-like gene *TaSPL16* in wheat (*Triticum aestivum* L.). Front. Plant Sci..

[49] Wang B.N., Geng S.F., Wang D., Feng N., Zhang D.D., Wu L., Hao C.Y., Zhang X.Y., Li A.L., Mao L. (2015). Characterization of squamosa promoter binding protein-like genes in wheat. J. Plant Biol..

[50] Lan T., Zheng Y., Su Z., Yu S., Song H., Zheng X., Lin G., Wu W. (2019). *OsSPL10*, a SBP-Box gene, plays a dual role in salt tolerance and trichome formation in rice (*Oryza sativa* L.). G3 Genes Genomes Genet..

[51] Zhang X.F., Yang C.Y., Lin H.X., Wang J.W., Xue H.W. (2021). Rice *SPL12* coevolved with *GW5* to determine grain shape. Sci. Bull..

[52] Shingate P., Ravi V., Prasad A., Tay B.H., Garg K.M., Chattopadhyay B., Yap L.M., Rheindt F.E., Venkatesh B. (2020). Chromosome-level assembly of the horseshoe crab genome provides insights into its genome evolution. Nat. Commun..

[53] Badaeva E.D., Dedkova O.S., Gay G., Pukhalskyi V.A., Zelenin A.V., Bernard S., Bernard M. (2007). Chromosomal rearrangements in wheat: Their types and distribution. Genome.

[54] Devos K.M., Atkinson M., Chinoy C., Francis H., Harcourt R., Koebner R., Liu C., Masojć P., Xie D., Gale M. (1993). Chromosomal rearrangements in the rye genome relative to that of wheat. Theor. Appl. Genet..

[55] Budak H., Akpinar B.A. (2015). Plant miRNAs: Biogenesis, organization and origins. Funct. Integr. Genom..

[56] Silva G.F.F.E., Silva E.M., da Silva Azevedo M., Guivin M.A.C., Ramiro D.A., Figueiredo C.R., Carrer H., Peres L.E.P., Nogueira F.T.S. (2014). *MicroRNA156*-targeted *SPL/SBP* box transcription factors regulate tomato ovary and fruit development. Plant J..

[57] Xu M., Hu T., Zhao J., Park M.Y., Earley K.W., Wu G., Yang L., Poethig R.S. (2016). Developmental Functions of *miR156*-regulated Squamosa promoter binding protein-like (SPL) genes in *Arabidopsis thaliana*. PLoS Genet..

[58] Zhang X., Zou Z., Zhang J., Zhang Y., Han Q., Hu T., Xu X., Liu H., Li H., Ye Z. (2011). Over-expression of sly-*miR156a* in tomato results in multiple vegetative and reproductive trait alterations and partial phenocopy of the *sft* mutant. FEBS Lett..

[59] Watanabe Y., Tomita M., Kanai A. (2007). Computational methods for *microRNA* target prediction. Methods Enzymol..

[60] Dai X., Zhao P.X. (2011). PsRNATarget: A plant small RNA target analysis server. Nucleic Acids Res..

[61] Borrill P., Ramirez-Gonzalez R., Uauy C. (2016). ExpVIP: A customizable RNA-seq data analysis and visualization platform. Plant Physiol..

[62] Ramirez-Gonzalez R.H., Borrill P., Lang D., Harrington S.A., Brinton J., Venturini L., Davey M., Jacobs J., van Ex F., Pasha A. (2018). The transcriptional landscape of polyploid wheat. Science.

[63] Baloglu M.C., Inal B., Kavas M., Unver T. (2014). Diverse expression pattern of wheat transcription factors against abiotic stresses in wheat species. Gene.

[64] Sharma S., Taneja M., Tyagi S., Singh K., Upadhyay S.K. (2017). Survey of high throughput RNA-Seq data reveals potential roles for lncRNAs during development and stress response in bread wheat. Front. Plant Sci..

[65] Evans M. (1974). Rapid responses to plant hormones. Annu. Rev. Physiol..

[66] Hongfei Q. (2008). Review of the research on plant stress resistance. J. Anhui Agric. Sci..

[67] Ning K., Chen S., Huang H.J., Jiang J., Yuan H.M., Li H.Y. (2017). Molecular characterization and expression analysis of the *SPL* gene family with *BpSPL9* transgenic lines found to confer tolerance to abiotic stress in *Betula platyphylla* Suk. Plant Cell Tissue Organ Cult..

[68] Chao L.M., Liu Y.Q., Chen D.Y., Xue X.Y., Mao Y.B., Chen X.Y. (2017). *Arabidopsis* transcription factors *SPL1* and *SPL12* confer plant thermotolerance at reproductive stage. Mol. Plant.

[69] Hu J., Huang L., Chen G., Liu H., Zhang Y., Zhang R., Zhang S., Liu J., Hu Q., Hu F. (2021). The elite alleles of *OsSPL4* regulate grain size and increase grain yield in rice. Rice.

[70] Qiao X., Li Q., Yin H., Qi K., Li L., Wang R., Zhang S., Paterson A.H. (2019). Gene duplication and evolution in recurring polyploidization-diploidization cycles in plants. Genome Biol..

[71] Salse J., Bolot S., Throude M., Jouffe V., Piegu B., Quraishi U.M., Calcagno T., Cooke R., Delseny M., Feuillet C. (2008). Identification and characterization of shared duplications between rice and wheat provide new insight into grass genome evolution. Plant Cell..

[72] Wang X., Yan X., Hu Y., Qin L., Wang D., Jia J., Jiao Y. (2022). A recent burst of gene duplications in *Triticeae*. Plant Commun..

[73] Xiong Y., Liu T., Tian C., Sun S., Li J., Chen M. (2005). Transcription factors in rice: A genome-wide comparative analysis between monocots and eudicots. Plant Mol. Biol..

[74] Yu J., Wang J., Lin W., Li S., Li H., Zhou J., Ni P., Dong W., Hu S., Zeng C. (2005). The Genomes of *Oryza sativa*: A history of duplications. PLoS Biol..

[75] Tang H., Wang X., Bowers J.E., Ming R., Alam M., Paterson A.H. (2008). Unraveling ancient hexaploidy through multiply-aligned angiosperm gene maps. Genome Res..

[76] Wicker T., Gundlach H., Spannagl M., Uauy C., Borrill P., Ramirez-Gonzalez R.H., De Oliveira R., Mayer K., Paux E., International Wheat Genome Sequencing Consortium (2018). Impact of transposable elements on genome structure and evolution in bread wheat. Genome Biol..

[77] Walkowiak S., Gao L., Monat C., Haberer G., Kassa M.T., Brinton J., Ramirez-Gonzalez R.H., Kolodziej M.C., Delorean E., Thambugala D. (2020). Multiple wheat genomes reveal global variation in modern breeding. Nature.

[78] Sato Y., Antonio B., Namiki N., Motoyama R., Sugimoto K., Takehisa H., Minami H., Kamatsuki K., Kusaba M., Hirochika H. (2011). Field transcriptome revealed critical developmental and physiological transitions involved in the expression of growth potential in japonica rice. BMC Plant Biol..

[79] Sato Y., Takehisa H., Kamatsuki K., Minami H., Namiki N., Ikawa H., Ohyanagi H., Sugimoto K., Antonio B.A., Nagamura Y. (2013). RiceXPro version 3.0: Expanding the informatics resource for rice transcriptome. Nucleic Acids Res..

[80] Xia L., Zou D., Sang J., Xu X., Yin H., Li M., Wu S., Hu S., Hao L., Zhang Z. (2017). Rice expression database (RED): An integrated RNA-Seq-derived gene expression database for rice. J. Genet. Genom..

[81] Altpeter F., Vasil V., Srivastava V., Vasil I.K. (1996). Integration and expression of the high-molecular-weight glutenin subunit 1Ax1 gene into wheat. Nat. Biotechnol..

[82] Blechl A.E., Anderson O.D. (1996). Expression of a novel high-molecular-weight glutenin subunit gene in transgenic wheat. Nat. Biotechnol..

[83] He G.Y., Rooke L., Steele S., Bekes F., Gras P., Tatham A.S., Fido R., Barcelo P., Shewry P.R., Lazzeri P.A. (1999). Transformation of pasta wheat (*Triticum turgidum* L. var. durum) with high-molecular-weight glutenin subunit genes and modification of dough functionality. Mol. Breed..

[84] Yao Q., Cong L., Chang J.L., Li K.X., Yang G.X., He G.Y. (2006). Low copy number gene transfer and stable expression in a commercial wheat cultivar via particle bombardment. J. Exp. Bot..

[85] Li T.D., Hu J.C., Sun Y., Li B.S., Zhang D.L., Li W.L., Liu J.X., Li D.W., Gao C.X., Zhang Y.L. (2021). Highly efficient heritable genome editing in wheat using an RNA virus and bypassing tissue culture. Mol. Plant.

[86] Wang Y., Cheng X., Shan Q., Zhang Y., Liu J., Gao C., Qiu J.L. (2014). Simultaneous editing of three homoeoalleles in hexaploid bread wheat confers heritable resistance to powdery mildew. Nat. Biotechnol..

[87] Wang K., Liu H., Du L., Ye X. (2017). Generation of marker-free transgenic hexaploid wheat via an Agrobacterium-mediated co-transformation strategy in commercial Chinese wheat varieties. Plant Biotechnol. J..

[88] Li Y., Fu X., Zhao M., Zhang W., Li B., An D., Li J., Zhang A., Liu R., Liu X. (2018). A Genome-wide view of transcriptome dynamics during early spike development in bread wheat. Sci. Rep..

[89] Ma S., Wang M., Wu J., Guo W., Chen Y., Li G., Wang Y., Shi W., Xia G., Fu D. (2021). WheatOmics: A platform combining multiple omics data to accelerate functional genomics studies in wheat. Mol. Plant.

[90] Pfeifer M., Kugler K.G., Sandve S.R., Zhan B., Rudi H., Hvidsten T.R., Mayer K.F., Olsen O.A. (2014). Genome interplay in the grain transcriptome of hexaploid bread wheat. Science.

[91] Li J., Tang B., Li Y., Li C., Guo M., Chen H., Han S., Li J., Lou Q., Sun W. (2021). Rice SPL10 positively regulates trichome development through expression of *HL6* and auxin-related genes. J. Integr. Plant Biol..

[92] Bai J.F., Wang Y.K., Wang P., Duan W.J., Yuan S.H., Sun H., Yuan G.L., Ma J.X., Wang N., Zhang F.T. (2017). Uncovering male fertility transition responsive miRNA in a wheat photo-thermosensitive genic male sterile line by deep sequencing and degradome analysis. Front. Plant Sci..

[93] Yan Y., Wei M., Li Y., Tao H., Wu H., Chen Z., Li C., Xu J. (2021). MiR529a controls plant height, tiller number, panicle architecture and grain size by regulating *SPL* target genes in rice (*Oryza sativa* L.). Plant Sci..

[94] Li J., Jiao Z., He R., Sun Y., Xu Q., Zhang J., Jiang Y., Li Q., Niu J. (2019). Gene expression profiles and *microRNA* regulation networks in tiller primordia, stem tips, and young spikes of wheat guomai 301. Genes.

[95] Wang M., Yang C., Wei K., Zhao M., Shen L., Ji J., Wang L., Zhang D., Guo J., Zheng Y. (2021). Temporal expression study of *miRNAs* in the crown tissues of winter wheat grown under natural growth conditions. BMC Genom..

[96] Li Y., Wang W., Ma C., Ming R. (2021). Editorial: Genomics-enabled crop genetics. Front. Genet..

[97] Finn R.D., Attwood T.K., Babbitt P.C., Bateman A., Bork P., Bridge A.J., Chang H.Y., Dosztányi Z., El-Gebali S., Fraser M. (2017). InterPro in 2017—Beyond protein family and domain annotations. Nucleic Acids Res..

[98] Letunic I., Bork P. (2018). 20 years of the SMART protein domain annotation resource. Nucleic Acids Res..

[99] Kumar S., Stecher G., Li M., Knyaz C., Tamura K. (2018). MEGA X: Molecular evolutionary genetics analysis across computing platforms. Mol. Biol. Evol..

[100] Larkin M.A., Blackshields G., Brown N.P., Chenna R., McGettigan P.A., McWilliam H., Valentin F., Wallace I.M., Wilm A., Lopez R. (2007). Clustal W and clustal X version 2.0. Bioinformatics.

[101] Chen C., Chen H., Zhang Y., Thomas H.R., Frank M.H., He Y., Xia R. (2020). TBtools: An integrative toolkit developed for interactive analyses of big biological data. Mol. Plant.

[102] Bailey T.L., Boden M., Buske F.A., Frith M., Grant C.E., Clementi L., Ren J., Li W.W., Noble W.S. (2009). MEME SUITE: Tools for motif discovery and searching. Nucleic Acids Res..

[103] Wang Y., Tang H., Debarry J.D., Tan X., Li J., Wang X., Lee T.H., Jin H., Marler B., Guo H. (2012). MCScanX: A toolkit for detection and evolutionary analysis of gene synteny and collinearity. Nucleic Acids Res..

[104] Tottman D., Makepeace R., Broad H. (1979). An explanation of the decimal code for the growth stages of cereals, with illustrations. Ann. Appl. Biol..

[105] Zadoks J.C., Chang T.T., Konzak C.F. (1974). A decimal code for the growth stages of cereals. Weed Res..

[106] Li X., Li Y., Yu X., Sun F., Yang G., He G. (2020). Genomics-enabled analysis of *puroindoline b2* genes identifies new alleles in wheat and related *Triticeae* species. Int. J. Mol. Sci..

[107] Supartana P., Shimizu T., Shioiri H., Nogawa M., Nozue M., Kojima M. (2005). Development of simple and efficient in planta transformation method for rice (*Oryza sativa* L.) using *Agrobacterium tumefaciens*. J. Biosci. Bioeng..

