# Supplementary material for: Conservation and Divergence of SQUAMOSA-PROMOTER BINDING PROTEIN-LIKE (SPL) Gene Family between Wheat and Rice
Source: Int J Mol Sci. 2022 Feb 14;23(4):2099. doi: 10.3390/ijms23042099 (PMC8874652; doi:10.3390/ijms23042099)
Supplement: Supplementary file 1 [file ijms-23-02099-s001.zip › WYS_IJMS_suppl/WYS_IJMS_Suppl_v5_20211214.pdf]

International Journal of *Molecular Sciences*

MDPI

Supplementary Information for

Article

Conservation and Divergence of *SQUAMOSA-PROMOTER BINDING PROTEIN-LIKE* (*SPL*) Gene Family between Wheat and Rice

Li Li †, Fu Shi †, Guoli Wang, Yanbin Guan, Yufan Zhang, Mingjie Chen, Junli Chang, Guangxiao Yang, Guangyuan He, Yuesheng Wang * and Yin Li *

The Genetic Engineering International Cooperation Base of Chinese Ministry of Science and Technology, Key Laboratory of Molecular Biophysics of Chinese Ministry of Education, College of Life Science and Technology, Huazhong University of Science and Technology, Wuhan 430074, China; hustll2017@hust.edu.cn (L.L.); hustshifu@hust.edu.cn (F.S.); m202071947@hust.edu.cn (G.W.); gyb@hust.edu.cn (Y.G.); d201980545@hust.edu.cn (Y.Z.); cmj@hust.edu.cn (M.C.); cjl@hust.edu.cn (J.C.); ygx@hust.edu.cn (G.Y.) ; hegy@hust.edu.cn (G.H.)

* Correspondence: wys@hust.edu.cn (Y.W.); yinli2021@hust.edu.cn (Y.L.)

† These authors contributed equally to this work.

Citation: Li, L.; Shi, F.; Wang, G.; Guan, Y.; Zhang, Y.; Chen, M.; Chang, J.; Yang, G.; He, G.; Wang, Y.; et al. Conservation and Divergence of *SQUAMOSA-PROMOTER BINDING PROTEIN-LIKE* (*SPL*) Gene Family between Wheat and Rice. *Int. J. Mol. Sci.* **2022**, *23*, 2099. https://doi.org/10.3390/ijms23042099

Academic Editor: Luigi Cattivelli

Received: 15 December 2021
Accepted: 30 January 2022
Published: 14 February 2022

Publisher's Note: MDPI stays neutral with regard to jurisdictional claims in published maps and institutional affiliations.

Copyright: © 2022 by the authors. Licensee MDPI, Basel, Switzerland. This article is an open access article distributed under the terms and conditions of the Creative Commons Attribution (CC BY) license (https://creativecommons.org/licenses/by/4.0/).

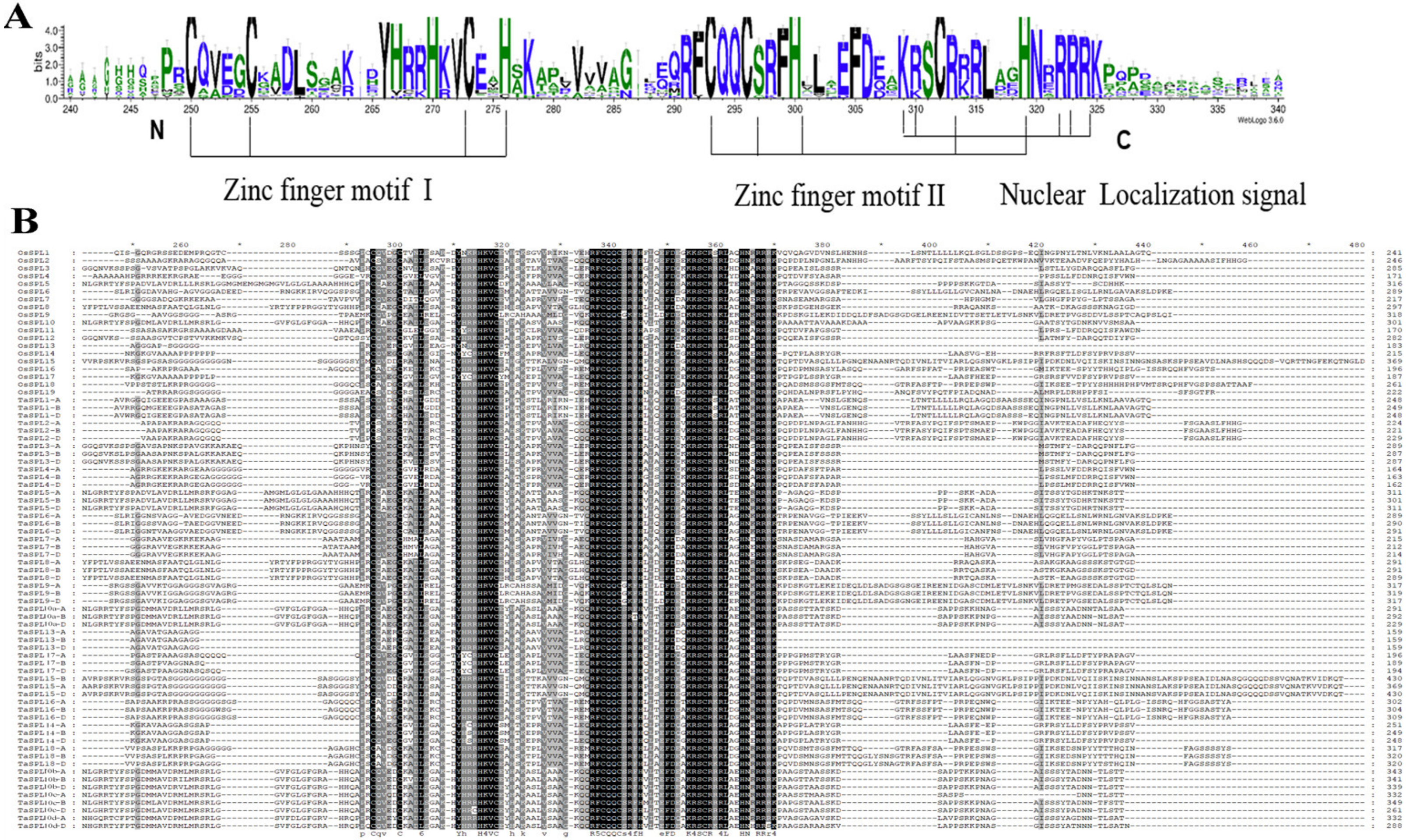

Figure S1. Multiple-sequence alignment of the TaSPL proteins. **(A)** Sequence logo analysis showing that the SBP domains among the TaSPL proteins share conserved zinc finger motifs I and II and nuclear localization signal. The overall height of each stacked amino acid residue represents the degree of conservation at this position, while the height of the letters within each stack indicates the relative frequency of the corresponding amino acid residues. **(B)** Multiple-sequence alignment of the 56 TaSPL and 19 OsSPL proteins, obtained using the ClustalW2.0 software.

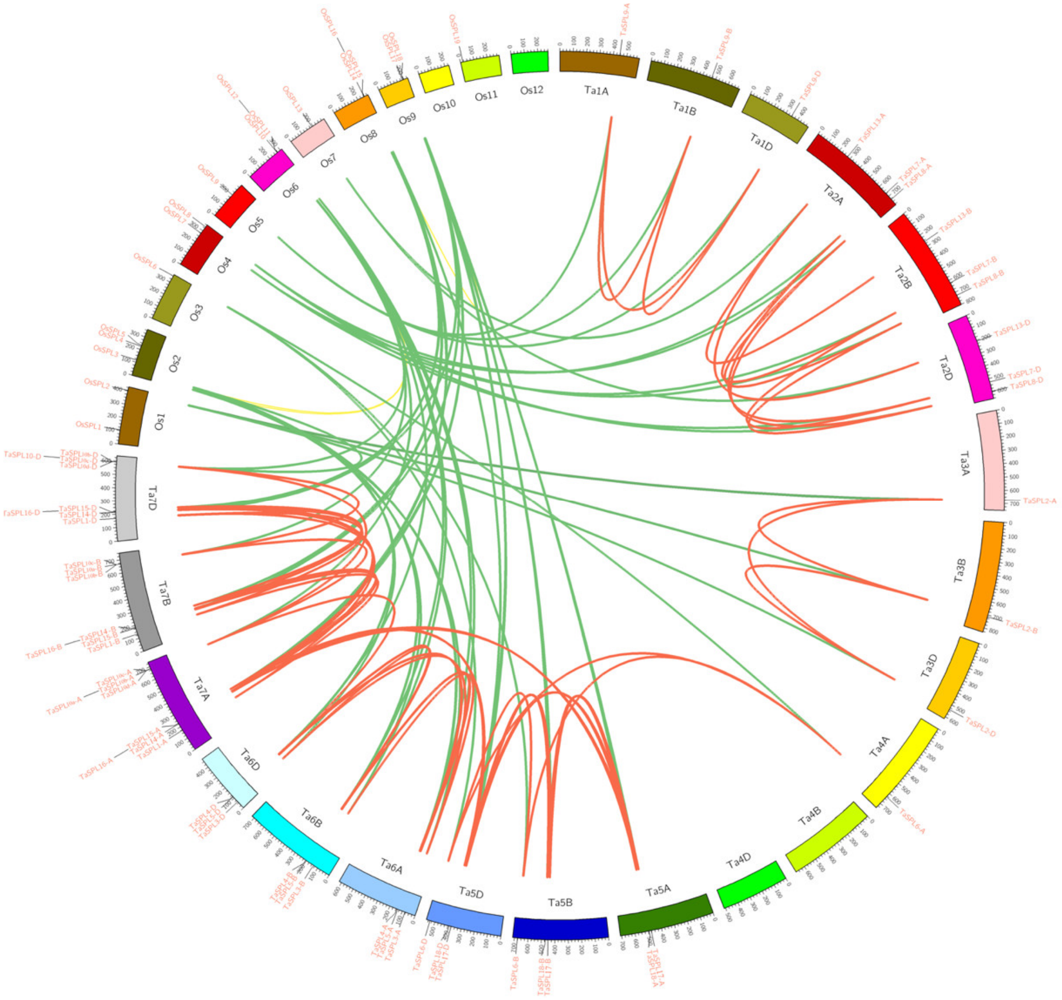

Figure S2. Syntenic analysis validating the orthologous relationship between *TaSPL* and *OsSPL* genes. The homeologous relationship within each triad confirmed by the syntenic analysis results is shown as red lines, while the orthologous relationships between *OsSPL*s and *TaSPLs* are shown as green lines, consistent with the phylogenetic results (**Figure 1A**).

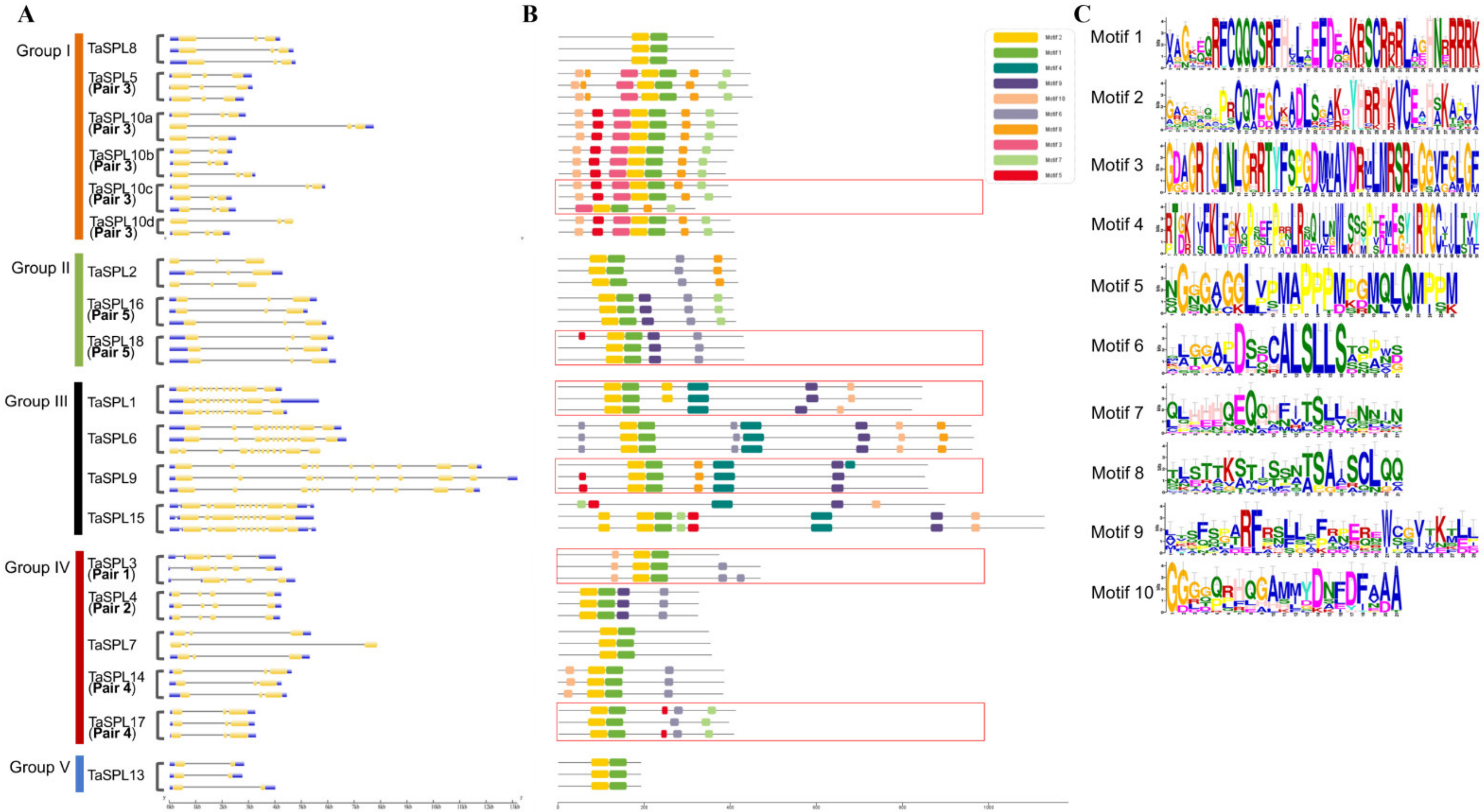

Figure S3. Gene structural and protein motif analyses emphasizing conservation of both gene and protein sequences between phylogenetically related *TaSPL* genes. The *TaSPL* genes and proteins were aligned first by phylogenetic groups and then by sub-genomes. **(A)** Analysis of exon–intron structures shows the *TaSPL* genes within each phylogenetic group share similar exon–intron structures. Exon–intron visualization was performed using the online tool GSDS [1]. Exons and introns are represented by yellow rectangles and grey lines, respectively. The blue rectangles indicate untranslated regions (UTRs). **(B)** Conserved protein motifs within *TaSPL*s were identified with MEME and visualized using TBtools [2]. This result not only shows that each phylogenetic group of *TaSPL*s has similar combinations of protein motifs, but also identifies different protein motifs (indicated by red boxes) between each group of homeologous copies, suggest-

ing sequence divergence. **(C)** The amino acid sequences of MEME-predicted motifs in *TaSPL*s. The position and length of these motifs can be identified, according to the scale bar, and the different motifs are represented by different colors.

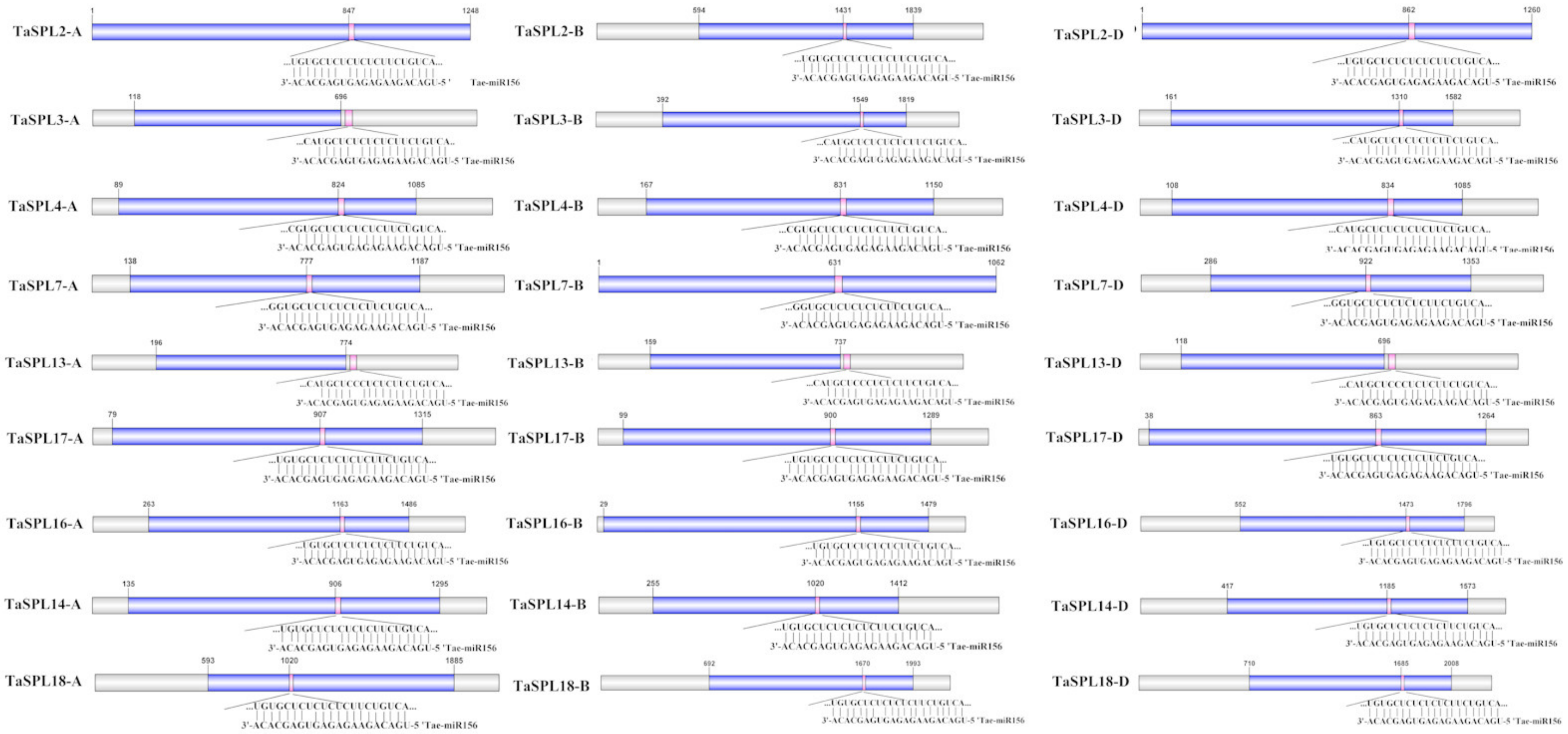

Figure S4. Possible miR156 cleavage sites in the *TaSPL* genes predicted by the psRNATarget server software [3]. The grey bars represent cDNA of the *TaSPL* genes, with the purple bars highlighting the open reading frames (ORFs). The red lines represent the predicted tae-miR156 cleavage sites, with the complementary cDNA and tae-miR156 sequences given in detail.

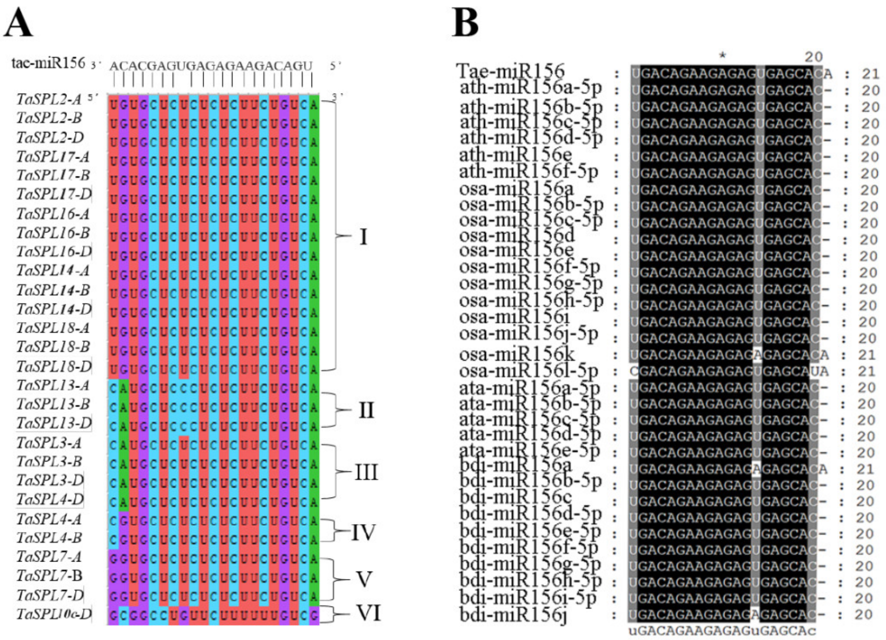

Figure S5. Conservation of the tae-miR156 cleavage site within the TaSPL genes (**A**) and the mature miR156 sequences between wheat, *Arabidopsis*, rice, *Ae. Tauschii,* and *Brachypodium distachyon* (*B. distachyon*) (**B**). (**A**) I to VI indicate different types of predicted tae-miR156 cleavage sequences in the 28 *TaSPL* genes. (**B**) Sequence alignment of mature miR156 from wheat, Arabidopsis, rice, *Ae. tauschii*, and *B. distachyon*. One mature tae-miR156 in wheat, six mature ath-miR156s in *Arabidopsis*, 12 mature osa-miR156s in rice, five mature ata-miR156s in *Ae. tauschii,* and ten mature bdi-miR516 in *B. distachyon,* obtained from miRBase [4], were used for sequence alignment.

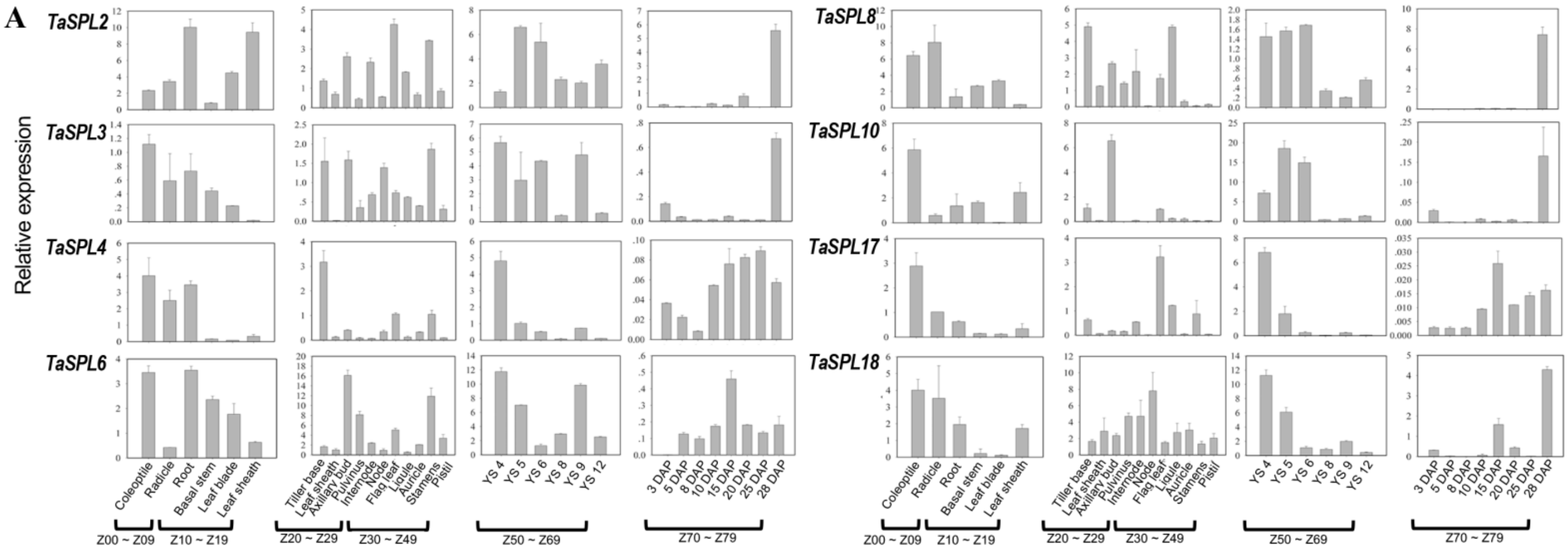

Figure S6. Quantitative PCR (qPCR) validation of spatial–temporal expression profiles of *TaSPL2/3/4/6/8/10/17/18* across developmental stages and tissues in wheat cv. China Spring. *TaActin* (TraesCS1B02G283900) was used as the reference gene. The various tissues and organs were collected following the scales of Zadoks and Tottman for cereal development processes [5, 6]. Z00–Z79 represent various vegetative and reproductive tissues at different developmental stages of wheat. Y-axis, relative expression levels; X-axis, different tissues; YS, young spikes; DAP, day after pollination.

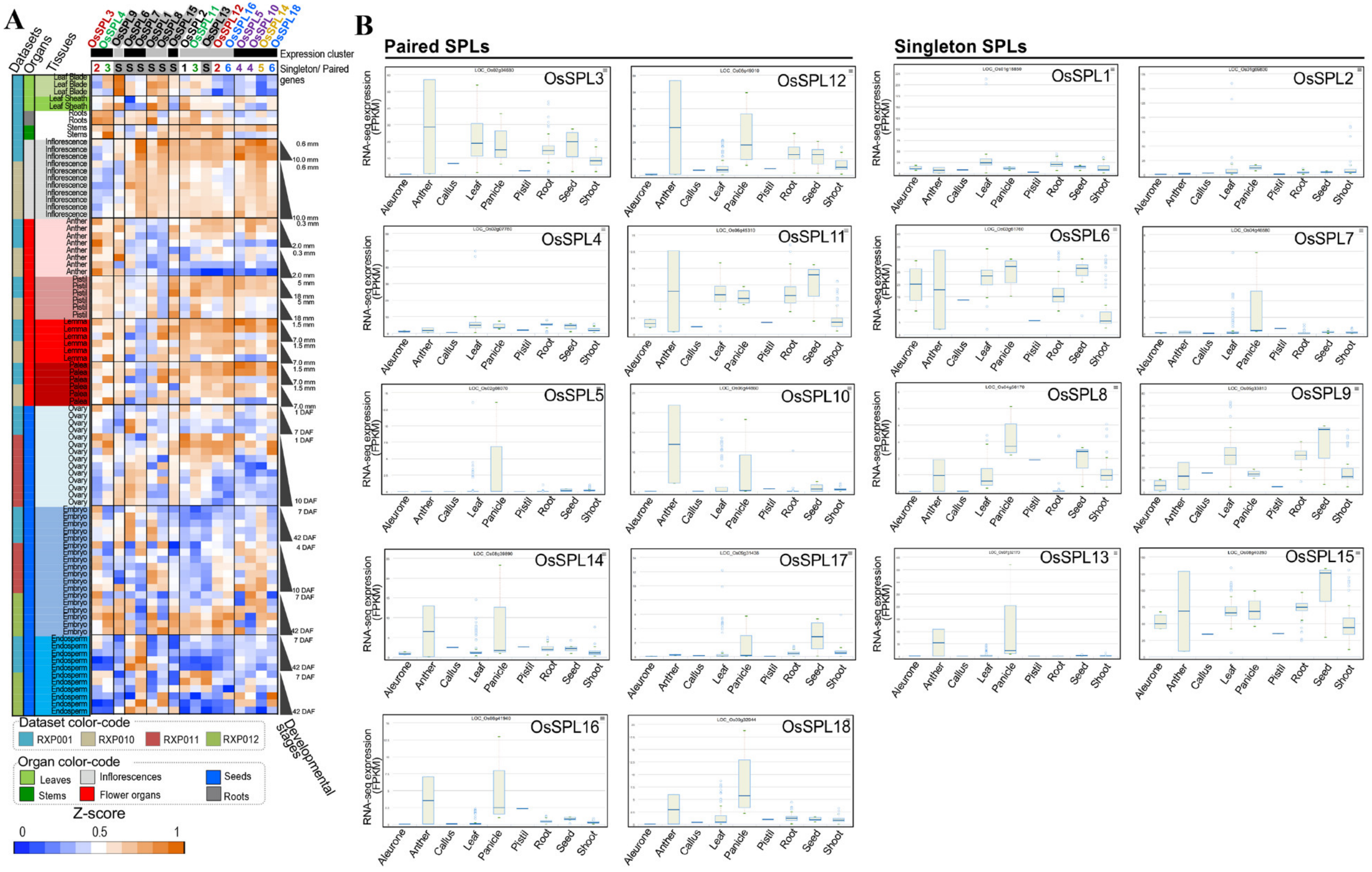

Figure S7. Expression profiles of *OsSPLs* based on microarray data (**A**) and RNA-seq data (**B**). (**A**) Microarray-based *OsSPL* expression profiles were retrieved from RiceXPro and visualized using heatmap. Each column represents an *OsSPL* gene, and each row represents a microarray sample, with the microarray data

sets, organs, and tissues labeled on the left, and the developmental stages labeled on the right of the heatmap. Information about microarray data sets and organs where the data were collected are color-coded. In Fig. S7A, singleton *OsSPL*s are shaded in grey, while evolutionarily paired *OsSPL*s are highlighted using colors, with red, green, gold, blue, and purple indicating Pair 1, Pair 2, Pair 3, Pair 4, and Pair 5, respectively. The *OsSPL* genes are row-clustered into seven clusters, based on expression similarity determined by k-means clustering. Abbreviations: DAF, days after flowering. (**B**) The RNA-seq-based expression abundance of each *OsSPL* gene was queried in the rice expression database (RED), and is shown in boxplots, in which gene expression values are normalized (in FPKM) between the RED data sets and grouped according to organs.

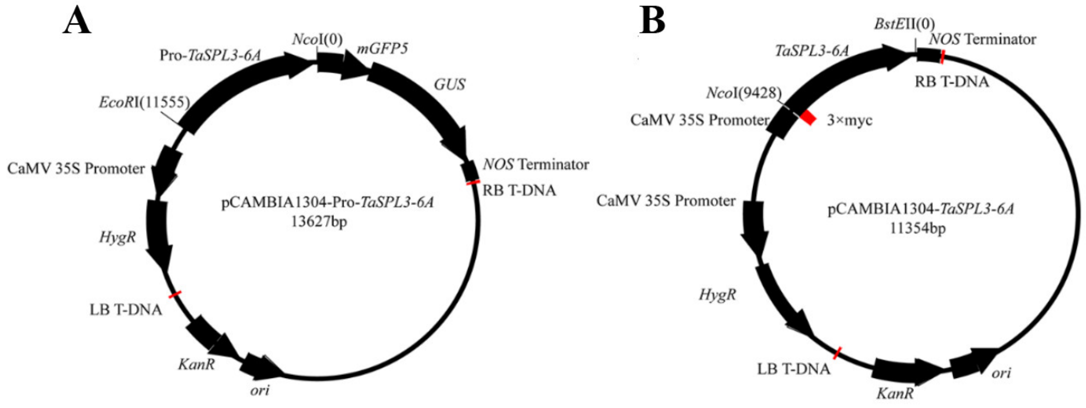

Figure S8. Diagrams of the promoter analysis vector (pCAMBIA1304-TaSPL3-6A-pro-GUS, **Fig. A**) and the overexpression vector (pCAMBIA1304-3 × myc-TaSPL3-6A, **Fig. B**). **(A)** In the pCAMBIA1304-TaSPL3-6A-pro-GUS vector, the TaSPL3-6A promoter (labeled as Pro-TSPL3-6A) drives the expression of a *GFP-GUS* fused gene (labeled as mGFP5 and GUS); **(B)** In the pCAMBIA1304-3 × myc-TaSPL3-6A vector, CaMV 35S promoter drives the expression of *TaSPL3-6A*, with its 5′ end fused to a 3x myc tag for detection.

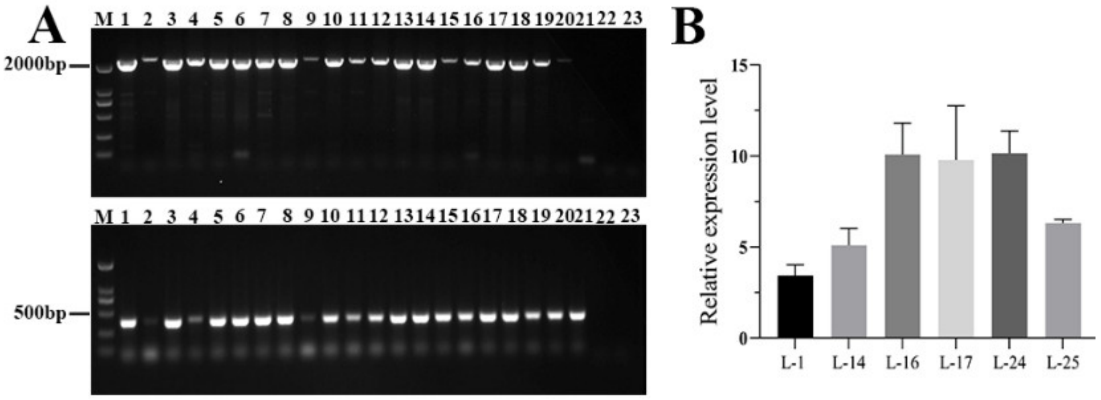

Figure S9. Molecular characterization of the TaSPL3-OE lines of rice. **(A)** PCR amplification of the *hygR* gene and the full sequence of the *TaSPL3-6A*. Lane M, DNA marker BM2000+. Lane 1, pCAMBIA1304-3 × myc-TaSPL3-6A for the positive control. Lanes 2–21, genomic DNA of the regenerated rice plants. Lane 22, genomic DNA of the negative control (rice cv. Nipponbare). Lane 23, negative PCR control using water as the template. The upper and lower panels were amplified by PCR using the full sequences of the *TaSPL3-6A* and *hygR* genes, respectively. **(B)** The transgenic lines were confirmed by a qRT-PCR method, where the primers were located in coding region of *TaSPL3-6A* gene with *OsActin* as the reference gene.

‘.

Table S1. Summary of the nomenclature for TaSPL genes from the present study and several previous studies [41-44 in the main text].

Gene Name_present study	geneID	proteinID	Chr.	No. of Exon	Protein length (aa)	Group*	Rice ortholog	Gene Name in Song et al. 2019 Agronomy	Gene Name in Zhu et al. 2020 BMC Plant Biol	Gene Name in Li et al. 2020 Sci Rep	Gene Name in Guo et al. 2020 Botany
TaSPL10a-A	TraesCS7A02G495100	TraesCS7A02G495100.1	chr7A	3	419	III	OsSPL10	TaSBP19-ALa	TaSPL041	TaSBP19A	TaSPL19A
TaSPL10a-B	TraesCS7B02G402200	TraesCS7B02G402200.1	chr7B	3	417	III	OsSPL10	TaSBP19-BLa	TaSPL046	TaSBP19D	TaSPL19B
TaSPL10a-D	TraesCS7D02G482500	TraesCS7D02G482500.1	chr7D	3	317	III	OsSPL10	TaSBP19-DLa	TaSPL056	na.	TaSPL19D
TaSPL13-A	TraesCS2A02G232400	TraesCS2A02G232400.1	chr2A	2	192	IV	OsSPL13	TaSBP2-ASa	TaSPL004	TaSBP2A	TaSPL2A
TaSPL13-B	TraesCS2B02G250900	TraesCS2B02G250900.1	chr2B	2	192	IV	OsSPL13	TaSBP2-BSa	TaSPL007	TaSBP2B	TaSPL2B
TaSPL13-D	TraesCS2D02G232800	TraesCS2D02G232800.1	chr2D	2	192	IV	OsSPL13	TaSBP2-DSa	TaSPL010	TaSBP2D	TaSPL2D
TaSPL17-A	TraesCS5A02G265900	TraesCS5A02G265900.1	chr5A	3	410	V	OsSPL17	TaSBP7-ALa	TaSPL017	na.	TaSPL7A
TaSPL17-B	TraesCS5B02G265600	TraesCS5B02G265600.1	chr5B	3	395	V	OsSPL17	TaSBP7-BLa	TaSPL019	TaSBP8B	TaSPL7B
TaSPL17-D	TraesCS5D02G273900	TraesCS5D02G273900.1	chr5D	3	407	V	OsSPL17	TaSBP7-DLa	TaSPL022	na.	TaSPL7D
TaSPL15-A	TraesCS7A02G249100	TraesCS7A02G249100.2	chr7A	9	898	II	OsSPL15	TaSBP14-ASa	TaSPL036	na.	TaSPL14A
TaSPL15-B	TraesCS7B02G142200	TraesCS7B02G142200.1	chr7B	10	1129	II	OsSPL15	TaSBP14-BSa	TaSPL043	TaSBP16B	TaSPL14B
TaSPL15-D	TraesCS7D02G248000	TraesCS7D02G248000.1	chr7D	10	1129	II	OsSPL15	TaSBP14-DSa	TaSPL051	TaSBP16D	TaSPL14D
TaSPL16-A	TraesCS7A02G260500	TraesCS7A02G260500.1	chr7A	3	407	I	OsSPL16	TaSBP15-ASa	TaSPL037	TaSBP15A	TaSPL15A
TaSPL16-B	TraesCS7B02G158500	TraesCS7B02G158500.1	chr7B	3	409	I	OsSPL16	TaSBP15-BSa	TaSPL045	TaSBP15B	TaSPL15B
TaSPL16-D	TraesCS7D02G261500	TraesCS7D02G261500.1	chr7D	3	414	I	OsSPL16	TaSBP15-DSa	TaSPL052	TaSBP15D	TaSPL15D
TaSPL14-A	TraesCS7A02G246500	TraesCS7A02G246500.1	chr7A	3	386	V	OsSPL14	TaSBP13-ASa	TaSPL035	TaSBP17A	TaSPL13A
TaSPL14-B	TraesCS7B02G144900	TraesCS7B02G144900.1	chr7B	3	386	V	OsSPL14	TaSBP13-BSa	TaSPL044	TaSBP17B	TaSPL13B
TaSPL14-D	TraesCS7D02G245200	TraesCS7D02G245200.1	chr7D	3	384	V	OsSPL14	TaSBP13-DSa	TaSPL050	TaSBP17D	TaSPL13D
TaSPL18-A	TraesCS5A02G286700	TraesCS5A02G286700.1	chr5A	3	430	I	OsSPL18	TaSBP8-ALa	TaSPL018	TaSBP7A	TaSPL8A
TaSPL18-B	TraesCS5B02G286000	TraesCS5B02G286000.1	chr5B	3	433	I	OsSPL18	TaSBP8-BLa	TaSPL020	TaSBP7B	TaSPL8B
TaSPL18-D	TraesCS5D02G294400	TraesCS5D02G294400.1	chr5D	3	432	I	OsSPL18	TaSBP8-DLa	TaSPL023	TaSBP7D	TaSPL8D
TaSPL1-A	TraesCS7A02G208000	TraesCS7A02G208000.1	chr7A	11	846	II	OsSPL1	TaSBP12-ASa	TaSPL034	na.	TaSPL12A
TaSPL1-B	TraesCS7B02G115200	TraesCS7B02G115200.1	chr7B	11	845	II	OsSPL1	TaSBP12-BSa	TaSPL042	TaSBP18B	TaSPL12B
TaSPL1-D	TraesCS7D02G210400	TraesCS7D02G210400.1	chr7D	11	822	II	OsSPL1	TaSBP12-DSa	TaSPL049	TASBP18D	TaSPL12D
TaSPL10b-A	TraesCS7A02G495000	TraesCS7A02G495000.1	chr7A	3	388	III	OsSPL10	TaSBP18-ALa	TaSPL040	TaSBP13A	TaSPL18A
TaSPL10b-B	TraesCS7B02G402300	TraesCS7B02G402300.1	chr7A	3	406	III	OsSPL10	TaSBP20-ALa	TaSPL047	TaSBP13B	TaSPL18B
TaSPL10b-D	TraesCS7D02G482400	TraesCS7D02G482400.1	chr7D	3	394	III	OsSPL10	TaSBP24-DLa	TaSPL055	TaSBP13D	TaSPL18D
TaSPL10c-A	TraesCS7A02G494900	TraesCS7A02G494900.1	chr7A	3	394	III	OsSPL10	TaSBP17-ALa	TaSPL039	TaSBP14A	TaSPL17A

TaSPL10c-B	TraesCS7B02G402400	TraesCS7B02G402400.1	chr7B	3	401	III	OsSPL10	TaSBP21-BLa	TaSPL048	TaSBP14B	TaSPL17B
TaSPL10c-D	TraesCS7D02G482300	TraesCS7D02G482300.1	chr7D	3	390	III	OsSPL10	TaSBP23-DLa	TaSPL054	TaSBP14D	TaSPL17D
TaSPL10d-A	TraesCS7A02G494800	TraesCS7A02G494800.1	chr7A	3	399	III	OsSPL10	TaSBP16-ALa	TaSPL038	TaSBP12A	TaSPL16A
TaSPL10d-D	TraesCS7D02G482200	TraesCS7D02G482200.1	chr7D	3	408	III	OsSPL10	TaSBP22-DLa	TaSPL053	TaSBP12D	TaSPL16D
TaSPL2-A	TraesCS3A02G432500	TraesCS3A02G432500.1	chr3A	3	415	I	OsSPL2	TaSBP5-ALa	TaSPL013	TaSBP5A	TaSPL5A
TaSPL2-B	TraesCS3B02G468400	TraesCS3B02G468400.1	chr3B	3	414	I	OsSPL2	TaSBP5-BLa	TaSPL014	TaSBP5B	TaSPL5B
TaSPL2-D	TraesCS3D02G425800	TraesCS3D02G425800.1	chr3D	3	419	I	OsSPL2	TaSBP5-DLa	TaSPL015	TaSBP5D	TaSPL5D
TaSPL3-A	TraesCS6A02G110100	TraesCS6A02G110100.1	chr6A	3	377	V	OsSPL3	TaSBP9-ASa	TaSPL025	TaSBP9A	TaSPL9A
TaSPL3-B	TraesCS6B02G138400	TraesCS6B02G138400.1	chr6B	4	473	V	OsSPL3	TaSBP9-BSa	TaSPL028	TaSBP9B	TaSPL9B
TaSPL3-D	TraesCS6D02G098500	TraesCS6D02G098500.1	chr6D	4	473	V	OsSPL3	TaSBP9-DSa	TaSPL031	TaSBP9D	TaSPL9D
TaSPL4-A	TraesCS6A02G155300	TraesCS6A02G155300.1	chr6A	4	328	V	OsSPL4	TaSBP11-ASa	TaSPL027	TaSBP10A	TaSPL11A
TaSPL4-B	TraesCS6B02G183400	TraesCS6B02G183400.1	chr6B	4	327	V	OsSPL4	TaSBP11-BSa	TaSPL030	TaSBP10B	TaSPL11B
TaSPL4-D	TraesCS6D02G145200	TraesCS6D02G145200.1	chr6D	4	325	V	OsSPL4	TaSBP11-DSa	TaSPL033	TaSBP10D	TaSPL11D
TaSPL5-A	TraesCS6A02G152000	TraesCS6A02G152000.1	chr6A	3	448	III	OsSPL5	TaSBP10-ASa	TaSPL026	TaSBP11A	TaSPL10A
TaSPL5-B	TraesCS6B02G180300	TraesCS6B02G180300.1	chr6B	3	442	III	OsSPL5	TaSBP10-BSa	TaSPL029	TaSBP11B	TaSPL10B
TaSPL5-D	TraesCS6D02G142100	TraesCS6D02G142100.1	chr6D	3	452	III	OsSPL5	TaSBP10-DSa	TaSPL032	TaSBP11D	TaSPL10D
TaSPL6-A	TraesCS4A02G359500	TraesCS4A02G359500.1	chr4A	11	960	II	OsSPL6	TaSBP6-ALa	TaSPL016	TaSBP6A	TaSPL6A
TaSPL6-B	TraesCS5B02G512800	TraesCS5B02G512800.1	chr5B	11	966	II	OsSPL6	TaSBP6-BLa	TaSPL021	na.#	TaSPL6B
TaSPL6-D	TraesCS5D02G513300	TraesCS5D02G513300.1	chr5D	12	962	II	OsSPL6	TaSBP6-DLa	TaSPL024	na.	TaSPL6D
TaSPL7-A	TraesCS2A02G413900	TraesCS2A02G413900.1	chr2A	3	349	IV	OsSPL7	TaSBP3-ALa	TaSPL005	TaSBP3A	TaSPL3A
TaSPL7-B	TraesCS2B02G432700	TraesCS2B02G432700.1	chr2B	3	353	IV	OsSPL7	TaSBP3-BLa	TaSPL008	na.	TaSPL3B
TaSPL7-D	TraesCS2D02G410700	TraesCS2D02G410700.1	chr2D	3	355	IV	OsSPL7	TaSBP3-DLa	TaSPL011	TaSBP3D	TaSPL3D
TaSPL8-A	TraesCS2A02G502300	TraesCS2A02G502300.1	chr2A	3	412	III	OsSPL8	TaSBP4-ALa	TaSPL006	TaSBP4A	TaSPL4A
TaSPL8-B	TraesCS2B02G530400	TraesCS2B02G530400.1	chr2B	3	408	III	OsSPL8	TaSBP4-BLa	TaSPL009	TaSBP4B	TaSPL4B
TaSPL8-D	TraesCS2D02G502900	TraesCS2D02G502900.1	chr2D	3	406	III	OsSPL8	TaSBP4-DLa	TaSPL012	TaSBP4D	TaSPL4D
TaSPL9-A	TraesCS1A02G255300	TraesCS1A02G255300.1	chr1A	10	859	II	OsSPL9	TaSBP1-ALa	TaSPL001	TaSBP1A	TaSPL1A
TaSPL9-B	TraesCS1B02G266100	TraesCS1B02G266100.1	chr1B	10	852	II	OsSPL9	TaSBP1-BLa	TaSPL002	TaSBP1B	TaSPL1B
TaSPL9-D	TraesCS1D02G254700	TraesCS1D02G254700.1	chr1D	10	859	II	OsSPL9	TaSBP1-DLa	TaSPL003	TaSBP1D	TaSPL1D

Note: * indicates the phylogenetic group reported in the present study; # "na.", not applicable.

Table S2. The sequences and purposes of the primers used in this study.

Primer name	Primer sequence (5′ to 3′)	Primer purpose	Plasmid
TaActin-F	AAAATATGGCATCACACGTTC	qRT-PCR	/
TaActin-R	ACACGAGCTACATCATCTAAGG	qRT-PCR	/
qTaSPL2F	ATGTTGCACGCGTCTAACAG	qRT-PCR	/
qTaSPL2R	AGTATCCATGGTACTGCTGCTG	qRT-PCR	/
qTaSPL3F	CATCATGGTTTCGATGGGTTC	qRT-PCR	/
qTaSPL3R	CAAGGCTCCCGTCGTCATT	qRT-PCR	/
qTaSPL4F	CAAGGCTCCCGTCGTCATT	qRT-PCR	/
qTaSPL4R	CATTGGTAGCAGCTCAGGTATGTC	qRT-PCR	/
qTaSPL6F	GGACAGACCACTGGCACCTAAT	qRT-PCR	/
qTaSPL6R	TGACTAAGTTGAAGCCTTCTACCCT	qRT-PCR	/
qTaSPL8F	TCAACATGGCTTCCCTTTCC	qRT-PCR	/
qTaSPL8R	AAGCCTGGCTGTTCTTGTTG	qRT-PCR	/
qTaSPL10F	TCTCCGACCAGAACAACAACAG	qRT-PCR	/
qTaSPL10R	ATCGGTCGTCTACATGAAGTCCAC	qRT-PCR	/
qTaSPL14F	GCAGCACCACCACCATCACA	qRT-PCR	/
qTaSPL14R	AACTCAGGCCGCCCAACGT	qRT-PCR	/
qTaSPL18F	TTCATGCTGTCAACCTGTGG	qRT-PCR	/
qTaSPL18R	TCACCGCGCAATGATTCTTG	qRT-PCR	/
qTaSPL3-6A-F	CATCATGGTTTCGATGGGTTC	qRT-PCR	/
qTaSPL3-6A-R	GGGGACTTGGCTGGAGGTT	qRT-PCR	/
T-TaSPL3-6A-F	CTCTGCATACCCTCTGTTGCG	Gene amplification	pMD-18-T-TaSPL3-6A
T-TaSPL3-6A-R	TTTTCCATGGCAACACGACT	Gene amplification	
1304-myc-TaSPL3-6A-F	ggggactcttgacc ATGGAACAAAAGTTGATCTCT	Plasmid construct	pCAMBIA1304-3×myc-TaSPL3-6A
1304-myc-TaSPL3-6A-R	aaattcgagctggtcac TCAGTGCATCCGGTCGAAGTG	Plasmid construct	
BD-TaSPL3-6A-N-F	ATATGGCCATGGAGGCCGAATTCATGGGCTCTTTTGGGATGGAG	Activation activity test	pGBKT7-TaSPL3-6A-N
BD-TaSPL3-6A-N-F	GGCCGCTGCAGGTCGACGGATCCTCAGTATGAGTTATGTGGCTTCTG	Activation activity test	
BD-TaSPL3-6A-SBP-F	ATATGGCCATGGAGGCCGAATTCTGTCAGGTTGAAGGCTGCAAAG	Activation activity test	pGBKT7-TaSPL3-6A-SBP
BD-TaSPL3-6A-SBP-R	GGCCGCTGCAGGTCGACGGATCCTCATGGCTTGCGCCTGCGGGAATTATG	Activation activity test	
BD-TaSPL3-6A-C-F	ATATGGCCATGGAGGCCGAATTCCAGCCAGAAGCAATTTCTTTCAGTTC	Activation activity test	pGBKT7-TaSPL3-6A-C
BD-TaSPL3-6A-C-R	GGCCGCTGCAGGTCGACGGATCCTCAGTGCATCCGGTCGAAGTG	Activation activity test	
BD-TaSPL3-6A-FL-F	ATATGGCCATGGAGGCCGAATTCATGGGCTCTTTTGGGATGGAGTG	Activation activity test	pGBKT7-TaSPL3-6A-FL

BD-TaSPL3-6A-FL-R	GGCCGCTGCAGGTCGACGGATCCTCAGTGCATCCGGTCGAAGT	Activation activity test	
pSGN-TaSPL3-6A-F	ttcatttggagagaacatctagaATGGGCTCTTTTG	Sub-cellular Localization	pSGN-TaSPL3-6A-GFP
pSGN-TaSPL3-6A-R	gtcgacagtactatcgatggatccGTGCATCCGGTC	Sub-cellular Localization	
1304-Test-F	GTACACAAATCGCCCGCAGA	Detection overexpression	/
1304-Test-R	CTTAATAACACATTGCGGACGTT	Detection overexpression	/
OsALL-F	CCGACAGTGGTCCCAAAGAT	Detection overexpression	/
OsALL-R	GCGCGCTATATTTTGTTTTCTATCG	Detection overexpression	/
qTaSPL3-6A-F	GAAGAGGACCTTGAGCAGAAATTG	Detection overexpression	/
qTaSPL3-6A-R	CTGGTTCCACTCCATCCCAA	Detection overexpression	/
OsTubulin-F	TACCGTGCCCTTACTGTTCC	Detection overexpression	/
OsTubulin-R	CGGTGGAATGTCACAGACAC	Detection overexpression	/
Pro-TaSPL3-6AF	CAAGTCCCCATCCTTGCAAATTAGGGAAGCC	Promoter amplification	
Pro-TaSPL3-6AR	GCCAGAGCCGAAGATCTCCGGCGCTCC	Promoter amplification	
1304-TaSPL3-6A-GUS-F	GCTATGACCATGATTACGAATTCCGATGTTCGTGAC	Plasmid construct	pCAMBIA1304-TaSPL3-6A-pro-GUS
1304-TaSPL3-6A-GUS-R	CTTTACTAGTCAGATCTACCATGGGCCAGAGCCGAAGAT	Plasmid construct	
Check-Pro-TaSPL3-6A-F	AACTTGATCTTTGGTCCCTC	Detection overexpression	
Check-Pro-TaSPL3-6A-R	GTTTTCGTCGGTAATCACCA	Detection overexpression	

Table S3. Summary of the expression patterns of *TaSPL*s in response to abiotic stress or phytohormone treatments. The references, methods for determining expression levels (qPCR or RNA-seq), cultivars analyzed, and treatment information are provided (as an EXCEL file).

Table S4. Analysis of yield parameters among transgenic TaSPL3-OE lines and control lines (wild-type Nipponbare and transgenic rice expressing the empty vector, VC) in rice.

Agronomic traits	WT	VC	L-1	L-14	L-16	L-17	L-24	L-25
Plant height (cm)	106.53±0.74 a*	105.96±2.97 a	88.90±2.20 c	93.46±1.04 bc	94.00±0.75 bc	81.06±1.10 d	93.80±3.65 bc	95.00±0.47 b
Length of spikes (cm)	25.50±0.50 a	25.33±0.25 a	22.78±0.42 b	22.42±0.49 b	22.62±0.40 b	22.51±0.74 b	22.75±0.51 b	22.52±0.21 b
Number of primary branches	11.11±0.42 a	10.83±0.30 a	9.57±0.42 b	10.66±0.40 ab	10.77±0.32 ab	9.40±0.67 b	10.55±0.47 ab	10.88±0.51 ab
Length of primary branches (cm)	10.45±0.22 a	10.20±0.30 a	9.80±0.25 b	8.40±0.13 d	9.06±0.18 c	10.13±0.24 ab	8.82±0.19 cd	8.47±0.16 cd
Number of secondary branches	30.11±1.61 a	25.25±2.39 a	17.71±2.59 c	19.88±1.82 bc	22.00±1.81 bc	24.40±2.80 ab	19.44±1.05 bc	24.66±1.91 ab
Weight per panicle (g)	4.02±0.11 a	3.66±0.14 a	2.26±0.13 c	2.52±0.11 bc	2.88±0.15 b	2.56±0.26 bc	2.53±0.09 bc	2.73±0.16 b
Number of grains per panicle	150.11±4.89 a	134.66±6 a	84.42±4.06 c	108.44±5.72 b	111.88±8.27 b	98.60±8.05 bc	97.55±4.66 bc	112.55±6.99 b
Number of vacant grains per panicle	13.44±2.16 cd	17.00±3.17 cd	26.28±5.31 b	11.88±1.29 d	16.77±2.59bcd	45.60±5.78 a	21.22±3.31bcd	22.55±2.16 bc
Weight of grains per panicle (g)	3.76±0.10 a	3.23±0.09 a	2.11±0.15 c	2.44±0.13 bc	2.63±0.15 b	2.23±0.24 bc	2.30±0.08 bc	2.51±0.15 bc
Tiller number	17.66±1.66	17.00±1.15	18.66±2.33	16.00±0.57	20.66±1.66	18.00±2.00	18.00±2.51	17.00±2.08
Thousand-grain weight (g)	25.09±0.41	24.38±0.55	25.11±0.55	23.16±0.91	23.73±0.58	23.72±1.14	23.60±0.68	23.74±0.20
Yield per plant (g)	66.61±3.96 a	51.16±0.78 a	42.49±4.10 bc	30.91±2.09 c	60.12±6.19 a	39.44±2.99 bc	40.25±1.66 bc	46.15±5.86 b
Length of flag leaf (cm)	58.00±2.64 a	54.33±1.20 a	41.66±0.88 b	36.33±0.66 c	40.33±0.88 bc	40.66±0.33 bc	44.56±2.43 b	41.33±0.33 b
Width of flag leaf (cm)	1.66±0.03 a	1.60±0.05 ab	1.50±0.00 bc	1.46±0.03 c	1.50±0.00 bc	1.53±0.03 bc	1.50±0.00 bc	1.43±0.06 b
Leaf area of flag leaf (cm2)	80.69±5.16 a	73.95±3.23 a	52.08±1.10 bc	44.44±1.80 c	50.41±1.10 bc	51.97±1.39 bc	55.70±3.04 b	49.33±1.91 bc

* Means with the same letter are not significantly different between the wild-type (cultivar Nipponbare), the vector control line, and the TaSPL3-OE transgenic lines ($P < 0.05$). All data are presented as the mean ± standard error of the mean.

Table S4. Summary of the functions of *OsSPL*s, determined using transgenic (including overexpression and RNAi), mutant, or CRISPR/Cas9-mediated knockout approaches (provided as an EXCEL file).

References

1. Hu, B.; Jin, J.; Guo, A. Y.; Zhang, H.; Luo, J.; Gao, G., GSDS 2.0: an upgraded gene feature visualization server. *Bioinformatics* **2015,** 31, 1296-7.
2. Chen, C.; Chen, H.; He, Y.; Xia, R., TBtools, a toolkit for biologists integrating various biological data handling tools with a user-friendly interface. *BioRxiv* **2018**, 289660.
3. Dai, X.; Zhao, P. X., psRNATarget: a plant small RNA target analysis server. *Nucleic Acids Res* **2011,** 39, W155-9.
4. Kozomara, A.; Griffiths-Jones, S., miRBase: integrating microRNA annotation and deep-sequencing data. *Nucleic Acids Res* **2011,** 39, D152-7.
5. Tottman, D.; Makepeace, R.; Broad, H., An explanation of the decimal code for the growth stages of cereals, with illustrations. *Annals of Applied Biology* **1979,** 93, 221-234.
6. Zadoks, J. C.; Chang, T. T.; Konzak, C. F., A decimal code for the growth stages of cereals. *Weed research* **1974,** 14, 415-421.
